# Inferred Subcellular Localization of Peroxisomal Matrix Proteins of *Guillardia theta* Suggests an Important Role of Peroxisomes in Cryptophytes

**DOI:** 10.3389/fpls.2022.889662

**Published:** 2022-06-16

**Authors:** Jana Vasilev, Ann-Kathrin Mix, Thomas Heimerl, Uwe G. Maier, Daniel Moog

**Affiliations:** ^1^Laboratory for Cell Biology, Department of Biology, Philipps-University Marburg, Marburg, Germany; ^2^Center for Synthetic Microbiology (SYNMIKRO), Philipps-University Marburg, Marburg, Germany

**Keywords:** peroxisomes, *Guillardia theta*, cryptophytes, *Phaeodactylum tricornutum*, ROS detoxification, β-oxidation, peroxisomal metabolism

## Abstract

Peroxisomes participate in several important metabolic processes in eukaryotic cells, such as the detoxification of reactive oxygen species (ROS) or the degradation of fatty acids by β-oxidation. Recently, the presence of peroxisomes in the cryptophyte *Guillardia theta* and other “chromalveolates” was revealed by identifying proteins for peroxisomal biogenesis. Here, we investigated the subcellular localization of candidate proteins of *G. theta* in the diatom *Phaeodactylum tricornutum*, either possessing a putative peroxisomal targeting signal type 1 (PTS1) sequence or factors lacking a peroxisomal targeting signal but known to be involved in β-oxidation. Our results indicate important contributions of the peroxisomes of *G. theta* to the carbohydrate, ether phospholipid, nucleotide, vitamin K, ROS, amino acid, and amine metabolisms. Moreover, our results suggest that in contrast to many other organisms, the peroxisomes of *G. theta* are not involved in the β-oxidation of fatty acids, which exclusively seems to occur in the cryptophyte's mitochondria.

## Introduction

Peroxisomes are essential but enigmatic compartments found in the majority of eukaryotes (Platta and Erdmann, 2007; Bolte et al., 2015; Reumann and Bartel, 2016; Moog et al., 2017). These single membrane-bound organelles are highly dynamic, not only with regard to their metabolic capacities, but also their organization, shape, and abundance in different organisms, tissues, and even cell types (Gabaldón, 2010; Hu et al., 2012). The most commonly observed peroxisomal functions are detoxification of reactive oxygen species (ROS) and β-oxidation of fatty acids (Pieuchot and Jedd, 2012). Beyond these, there is huge plasticity in peroxisome biochemistry across eukaryotic diversity.

Although peroxisomes vary drastically in protein content, the machinery for biogenesis, division, and maintenance is strictly conserved (Michels et al., 2005). These processes are facilitated by the so-called peroxins (Pex), for which a conserved core set can be found in each peroxisome-containing organism described so far (Gabaldón et al., 2006; Schlüter et al., 2006; Moog et al., 2017). This fact clearly points to a single evolutionary origin of peroxisomes in eukaryotes (Pieuchot and Jedd, 2012; Bolte et al., 2015). The conserved set of peroxins constitutes the peroxisomal importomer – the translocation machinery on the cytosolic face of the organelle and in the peroxisomal membrane (Meinecke et al., 2010). Similar to the peroxisomal import machinery, targeting signals of proteins destined for import into peroxisomes are highly conserved. Such proteins, if soluble, contain either a peroxisomal targeting signal 1(PTS1) consisting of a C-terminal tripeptide or a PTS2 signal, which resembles a N-terminal nonapeptide (Petriv et al., 2004; Kim and Hettema, 2015; Reumann et al., 2016). Whereas PTS1 is recognized by the signal-specific receptor Pex5, the more rare PTS2 signal-containing proteins are imported *via* Pex7-mediated translocation (Smith and Aitchison, 2013; Platta et al., 2014; Reumann and Bartel, 2016).

Remarkably, and in contrast to most other translocation systems in eukaryotic cells, the peroxisomal importomer, which at least in parts shares common ancestry with the ER-associated protein degradation machinery (ERAD) (Gabaldón et al., 2006; Schlüter et al., 2006; Schliebs et al., 2010; Bolte et al., 2011; Kienle et al., 2016), is able to transport folded proteins and even oligomeric substrates (Erdmann and Schliebs, 2005; Schrader and Fahimi, 2008). This is the reason why some proteins without detectable PTS can be transported by the so-called piggyback mechanism into the peroxisomal lumen.

The conservation of cell biological traits on the one hand and the metabolic variation on the other shows the adaptability of peroxisomes in different taxa, which is beneficial to organisms responding to changing environmental conditions. Peroxisomes have clearly played an important role in the evolution of eukaryotic cells (Bolte et al., 2015). In multicellular eukaryotes such as mammals, peroxisomes are of critical importance: peroxisomal disorders are linked to several severe human diseases (Schrader and Fahimi, 2008; Smith and Aitchison, 2013). The extent to which peroxisomes are essential in other eukaryotes can only be guessed based on what has been determined for a handful of laboratory model organisms. These include animals, higher plants (Arabidopsis), and fungi such as yeast, whereas the majority of eukaryote diversity – predominantly single-celled organisms – has not been studied in detail (Gabaldón, 2010). Recently, a unicellular green alga became an important key player in peroxisomal research. It was shown that *Chlamydomonas reinhardtii* imports proteins containing typical and non-canonical PTS into peroxisomes (Hayashi and Shinozaki, 2012; Lauersen et al., 2016; Kato et al., 2021). Moreover, peroxisomes of *C. reinhardtii* are involved in several important metabolic processes such as the β-oxidation of fatty acids, the glyoxylate cycle, and H_2_O_2_ detoxification (Lauersen et al., 2016; Kong et al., 2017; Kato et al., 2021).

Microalgae include some of the most ecologically significant and abundant primary producers on earth. Some of them evolved by an endosymbiosis between two eukaryotic cells and have thus a highly complex subcellular compartmentalization. More specifically, they contain complex plastids surrounded by three or four membranes that stem from eukaryotic endosymbionts (red or green algae). One of them is the cryptophyte *Guillardia theta*, which contains a complex plastid of red algal ancestry. A unique feature of this organism is that its complex four membrane-bound plastid still maintains a remnant of the nucleus of the former red algal endosymbiont – the nucleomorph – between the two outer and two inner plastidial membrane pairs (Archibald, 2015). Although evolutionarily and ecologically important organisms, less is known about the presence and function of peroxisomes in microalgae.

Molecular biological studies on peroxisomes of exotic microalgae such as cryptophytes are rare and have only recently been initiated on a genome wide scale (Moog et al., 2017; Ludewig-Klingner et al., 2018; Mix et al., 2018). Recently, we have identified a complete set of peroxins in the cryptophyte *G. theta*, strongly indicating the presence of peroxisomes in these evolutionarily significant microalgae (Mix et al., 2018). Similar observation was made for other chromalveolates (organisms with complex plastids of red algal origin) including haptophytes, stramenopiles, and alveolates, the latter including apicomplexan parasites, which are supposed to lack peroxisomes, chromerids, and dinoflagellates (Moog et al., 2017; Mix et al., 2018; Ludewig-Klingner et al., 2018). In diatoms, not only the presence of peroxins was investigated, but also peroxisomal metabolic functions were studied *via* initial localization studies and bioinformatic predictions suggesting that diatom peroxisomes contain enzymes for fatty acid β-oxidation, ROS detoxification, and the glyoxylate cycle (Kroth et al., 2008; Gonzalez et al., 2011), but almost no enzymes for photorespiration (Davis et al., 2017), which are typical for peroxisomes of other phototrophs such as plants (Reumann and Bartel, 2016).

Our previous work in *G. theta* strongly implies that peroxisomes exist in these secondarily evolved algae, which – in contrast to, e.g., diatoms – appear to be capable of PTS1- and PTS2-dependent protein import. However, less is known about the metabolic, cell biological, and evolutionary significance of peroxisomes in cryptophytes. Here, we report on the putative metabolic functions and cell biology of peroxisomes in the cryptophyte *G. theta*.

## Materials and Methods

### *In silico* Analyses/Bioinformatics

*Guillardia theta* proteins (Guith1, gene catalog, and best model proteins, https://mycocosm.jgi.doe.gov/Guith1/Guith1.home.html) were used for the identification of PTS1-containing proteins *via* search for the C-terminal tripeptide with the consensus sequence [SAC]-[KRH]-[LM] using a local script. Identified candidates were analyzed *via* KOALA BLAST in the Kyoto Encyclopedia of Genes and Genomes (KEGG; https://www.kegg.jp/blastkoala/), and these sequences were additionally used as queries for BLASTP analyses in NCBI (https://blast.ncbi.nlm.nih.gov/Blast.cgi?PAGE=Proteins). Putative PTS1-containing candidates were further analyzed with localization prediction tools for signal peptides (SignalP 3.0 (https://services.healthtech.dtu.dk/service.php?SignalP-3.0) and SignalP 4.1 (https://services.healthtech.dtu.dk/service.php?SignalP-4.1)), mitochondrial targeting peptides (TargetP v1.1 (https://services.healthtech.dtu.dk/service.php?TargetP-1.1), Predotar (https://urgi.versailles.inra.fr/predotar/), PredSL (http://aias.biol.uoa.gr/PredSL/input.html)), and PTS1 (http://mendel.imp.ac.at/pts1/PTS1predictor.jsp) and were additionally screened for transmembrane domains (http://www.cbs.dtu.dk/services/TMHMM/).

For the identification of factors involved in β-oxidation known protein sequences of *Phaeodactylum tricornutum, Arabidopsis thaliana* or *Saccharomyces cerevisiae* were used as queries in BLAST analyses. Identified putative sequences were analyzed bioinformatically as described above. For further differentiation, candidates for Gt_ACOX/Gt_ACAD with the ID_1477553/ID_90066 were bioinformatically analyzed in PHYRE^2^ (Kelley et al., 2015; http://www.sbg.bio.ic.ac.uk/phyre2/html/page.cgi?id=index) and a protein data bank (https://www.rcsb.org/).

Putative PTS2-proteins in *G. theta* were identified using an in-house script searching for the nonapeptide consensus [RK]-[LVIQ]-X_5_-[HQ]-[LAF] within the first 50 amino acids. Furthermore, some identified protein sequences that might be located and connected to *in vivo* localized PTS1 proteins from the first approach were analyzed manually according to the presence of a PTS2-like signal.

### Culture Conditions

The diatom *P. tricornutum* (Bohlin, UTEX646) was cultured in 500-ml Erlenmeyer flasks at 21°C under constant shaking (150–200 rpm) and constant light (24 h; 8,000–10,000 Lux) in 150 ml liquid f/2 medium (pH 8.0) containing 1.66% (w/v) Tropic Marin (Dr. Biener GmbH) salt, 2 mM Tris/HCl (pH 8.0), and 1.5 mM NH_4_Cl as a nitrogen source, or on solid f/2 plates containing agar (1,5% w/v). For the selection of positive transfected clones, Zeocin (InvivoGen) was added to a final concentration of 75 μg/ml. Protein overexpression was induced using f/2 medium with 0.89 mM NaNO_3_ as nitrogen source in 50 μl liquid f/2 medium for 24 h or on solid f/2 agar plates for 72 h as described before (Mix et al., 2018).

The cryptophyte *G. theta* (CCAM2327/CCMP2712) was cultured at 21°C in stationary Erlenmeyer flasks in a daily 14-h light and 10-h dark cycle (ca. 750 Lux) in 200 ml f/2 liquid medium (pH 7.2) containing 3.0% (w/v) Tropic Marin (Dr. Biener GmbH) salt, 5 mM Tris/HCl (pH 8.0), and 500 mM NH_4_Cl as a nitrogen source.

### DNA/RNA Isolation and cDNA Synthesis

For DNA/RNA isolation, *G. theta* was cultured in 200 ml f/2 medium for 7–14 days, as described earlier (Mix et al., 2018). *G. theta* cells were centrifuged at 3,000 x g and 21°C for 10 min. Total DNAs were isolated from the pelletized *G. theta* cells *via* the CTAB method and were stored at −20°C. Total RNAs of *G. theta* were extracted using the RNeasy Mini kit (Qiagen) and were promptly treated with DNAseI (Thermo Fisher Scientific), following the manufacturer's instructions, to remove potential genomic DNA contamination. cDNA was synthesized with the FastGene Scriptase II Kit (Nippon Genetics) using random hexamer oligonucleotides provided by the manufacturer. Synthesized cDNA was used for amplification of gene sequences with specific oligonucleotides (refer to Supplementary Table S4).

### Plasmid Construction and Biolistic Transformation of *P. tricornutum* Cells

For localization studies, selected candidate genes were amplified *via* standard polymerase chain reaction (PCR) on synthesized *G. theta* cDNA using the Q5^®^ High-Fidelity DNA Polymerase (NEB) and gene-specific oligonucleotides synthesized by MERCK (Germany). Amplified PCR fragments were subcloned into pJet1.2/blunt vector using the CloneJET™ PCR Cloning Kit (Thermo Fisher Scientific), sequenced (Macrogen), and finally fused to the enhanced green fluorescence protein (GFP) into the pPhaNR vector (GenBank: JN180663) *via* specific restriction sites. Generated plasmids were purified from a 50 ml *E. coli* TOP10 culture using the NucleoBOND^®^ MIDI Kit (Macherey-Nagel) and verified by sequencing (Macrogen). Tungsten M10 particles were coated with 5 μg of plasmid DNA and were used for biolistic transformation of *P. tricornutum* wildtype or mRuby3-SKL expressing strain with the Biolistic PDS-1000/He Particle Delivery System (Bio-Rad) using 1,350 psi rupture discs, as described previously (Apt et al., 1996).

### Protein Extraction, SDS-PAGE, and Western Blot Analyses

A 2-week-old *G. theta* wildtype culture was used for protein extraction. The cells were harvested and washed with PBS buffer before lysis with an extraction buffer containing 125 mM Tris-HCl (pH 8), 3 % (v/v) SDS, 0.2 % Triton X-100, and 0.5 % (v/v) proteinase inhibitor cocktail (PIC). The proteins were precipitated 30 min on ice with trichloroacetic acid at final concentration of 10% (v/v). After precipitation, the proteins were washed at least three times with 80 % (v/v) acetone, dried, and resuspended in 2x urea buffer. Approximately 15 μg of total protein extract was loaded on a sodium dodecyl sulfate-polyacrylamide gel electrophoresis (SDS-PAGE), separated, and transferred on a nitrocellulose membrane using a Pierce Semi-Dry Blotter (Thermo Fisher Scientific). Immunodetection was performed with a specific anti-peptide antibody raised against the last 12 amino acids of the putative urate oxidase in *G. theta* (α-Gt_UO.12aaC, 1:3000, David's Biotechnology).

### Confocal Laser Scanning Microscopy

Gene expression in *P. tricornutum* was induced for 24 h in 50 μl f/2 medium containing 0.89 mM NaNO_3_ as nitrogen source (induction of nitrate reductase promoter in pPhaNR) or on solid f/2 plates for 72 h. To monitor the expressed proteins fused to GFP Leica TCS SP2 or TCS SP5 confocal laser scanning microscopes and an HCX PL APO 40_/1.25–0.75 Oil CS or HCX PL APO 40x/1.25-0.75 Oil Lbd. bl., objectives were used. The 65 or 100 mW Argon laser was used to excite the GFP fluorescence and chlorophyll autofluorescence at 488 nm with emission spectra of 500–520 nm for GFP and 625–720 nm for the plastid autofluorescence, respectively. The peroxisomal marker, mRuby3-SKL, was excited at 543 nm with the HeNe 1.2 mW laser at TCS SP2 or at 561 nm with DPSS 10 mW 561 nm at TCS SP5, and emission was detected in a range of 580–605 nm (Marter et al., 2020).

### Electron Microscopy

*P. tricornutum* clones expressing GFP-Gt_UO were further analyzed using the transmission electron microscopy (TEM). Therefore, 1-week-old liquid cultures (under non-inducing conditions) were induced for 24 h with 0.89 mM NaNO_3_. The cells were harvested (centrifugation at 1,500 × g, 21°C, 5 min) and were frozen by high pressure (Wohlwend HPF Compact 02). After subsequent freeze substitution (with acetone, containing 0.25% osmium tetroxide, 0.2% uranyl acetate, 0.05% ruthenium red, and 5% water) (Leica AFS2), the cells were embedded in Epon812 substitute resin (Fluka). Embedded cells were sectioned to 60-nm-thin sections with Leica EM UC7 RT, which were used for immunolabeling with α-GFP (Rockland; dilutions 1:500 and 1:1,000) or a specific anti-peptide antibody (α-Gt_UO.12aaC) against the last C-terminal 12 amino acids of Gt-UO provided by David's Biotechnology (dilutions 1:200, 1:500, and 1:1000). Additionally, ultrathin sections of *G. theta* wildtype cells were used for immunolabeling with α-Gt_UO.12aaC. As a secondary antibody, goat-anti-rabbit and rabbit-anti-goat antibodies coupled to ultrasmall gold particles were used, respectively (Aurion, dilution 1:100). Subsequently, a silver enhancement procedure was done (Danscher, 1981) and sections were post-stained with 2% uranyl acetyte and 0.5% lead citrate. Analysis of the samples was conducted with a JEOL JEM2100 TEM equipped with a fast-scan 2k CCD TVIPS (Gauting, Germany) F214 camera.

## Results

### Bioinformatic Analyses of PTS1-Containing Proteins in *G. theta*

Less is known about the existence and function of peroxisomes in cryptophytes. Recently, the existence of a complete core set of peroxins (Pex) responsible for peroxisome biogenesis and maintenance was reported in *G. theta*, indicating the presence of peroxisomes in the cryptophyte (Mix et al., 2018). Here, we investigated the *G. theta* proteome regarding peroxisomal metabolism with the focus on PTS1-containing proteins. The most peroxisomal proteins reported so far contain the PTS1 signal (Gould et al., 1989; Brocard and Hartig, 2006), which is more abundant and conserved than the N-terminal nonapeptide PTS2. Besides, localization studies of PTS1-containing proteins can be conducted in *P. tricornutum* (Gonzalez et al., 2011; Mix et al., 2018).

A total of 24,840 nucleus-encoded *G. theta* proteins revealed by genome sequencing (Curtis et al., 2012) were analyzed for the presence of the C-terminal PTS1 signal consisting of [SAC]-[KRH]-[LM] within the last three amino acids of each sequence using an in-house script. The detected protein sequences were further analyzed *via* BLAST (refer to Materials and Methods). In total, 64 candidates were identified containing a putative PTS1 signal which were further analyzed with the PTS1 predictor (refer to Materials and Methods) to approve the character of the assumed PTS1 sequence. Thereby, for 58 of 64 putative PTS1 candidates (= 90.6%), the predicted PTS1 signal was confirmed (refer to Supplementary Table S1). Additionally, all PTS1-predicted proteins were screened for N-terminal targeting signals and transmembrane domains to identify potential false-positive candidates and sequences with a putative dual targeting. Using BlastKOALA, 1/3 of the 64 putative peroxisomal proteins were annotated and covered several essential metabolic functions, indicating the importance of the cryptomonad's peroxisomes within the cellular metabolic network in *G. theta* (refer to Supplementary Table S1). In summary, 31 out of 58 (53.44 %) predicted PTS1-containing proteins were identified to play a role in *G. theta*'s peroxisomal metabolism, and 19 of the 31 (61.3 %) proteins were predicted without any additional targeting signals or transmembrane domains (refer to Supplementary Table S1).

Although the typical peroxisomal enzyme catalase is usually a significant factor in the cellular detoxification of H_2_O_2_, no homolog *G. theta* protein was detected. Furthermore, no factors involved in β-oxidation of fatty acids, photorespiration, or glyoxylate cycle were identified in the cryptomonad containing a classical PTS1 signal, which is, for example, contrary to diatoms *P. tricornutum* and *Thalassiosira pseudonana*, (Gonzalez et al., 2011; Davis et al., 2017), indicating a special role of peroxisomes in the cryptomonad. Nevertheless, *in silico* results proposed the existence of several classical peroxisomal enzymes: ascorbate peroxidase (APX), urate oxidase (UO), 1,4-dihydroxy-2-naphthoyl-CoA synthase (MenB), alkyldihydroxyacetonephosphate synthase (AGPS) that participate in ROS detoxification, purine and ether phospholipid metabolisms, and vitamin K synthesis (refer to Table 1). Additionally, enzymes involved in carbohydrate, amine, and amino acid metabolism were identified containing a putative PTS1 signal at the C-terminus (refer to Table 1). Although *G. theta* contains the PTS2 receptor Pex7 and the corresponding Pex5 as potential co-receptor with a Pex7-binding domain (Mix et al., 2018), bioinformatic analyses using an in-house script revealed only very few potential PTS2-containing candidates (refer to Supplementary Table S2) with the consensus [RK]-[LVIQ]-X_5_-[HQ]-[LAF] present within the first 50 amino acids (Osumi et al., 1991; Swinkels et al., 1991; Petriv et al., 2004; Rucktäschel et al., 2011). The most promising PTS2-candidate in *G. theta* is a homolog to the mevalonate kinase (MVK) with a putative PTS2 signal at the N-terminus consisting of “KLILFGEHF,” which participates in isoprenoid biosynthesis.

**Table 1:** Putative peroxisomal PTS1(like)-containing proteins of *G. theta* were heterologously localized in *P. tricornutum*.

**Protein ID JGI**	**Abb**.	**Function (BLASTP)**	**Metabolism**	**PTS1**	**GFP-FL**	**GFP-12aaC**	**FL-GFP**
88523	Gt_APX	ascorbate peroxidase	ROS	SKM	**Px**	**Px**	nd
85732	Gt_MenB	1,4-dihydroxy-2-naphthoyl-CoA synthase	vitamin K	SRL	**Px**	**Px**	**C**
161824	Gt_UO	urate oxidase	purine	SKL	**Px**	**Px**	**C**
67814	Gt_ALN	allantoinase	purine	AKL	**C**	**Px**	**nd**
71482	Gt_ALC	allantoicase	purine	SKV^*^	**C**	**C**	**nd**
64327	Gt_MD	malate dehydrogenase	carbohydrate	SKL	**Px**	nd	nd
91319	Gt_NME	NAD-dependent malic enzyme	carbohydrate	CKL	**Px**	nd	nd
135385	Gt_FAR	fatty acyl-CoA reductase1-like	ether phospholipid	SRL	**Px**	nd	nd
157810	Gt_AGPS	alkyldihydroxyacetonephosphate synthase	ether phospholipid	SHL	**C**	**C/Px**	nd
158574	Gt_AAT	aspartate-amino transferase	amino acid	SKL	**C**	**Px**	nd
122115	Gt_MAO	monoamine oxidase	amine	SRL	nd	**Px**	nd

*Putative PTS1-cotaining proteins in G. theta (https://mycocosm.jgi.doe.gov/Guith1/Guith1.home.html) were identified in an in-house script and the C-terminal 12 amino acids were verified with the PTS1 predictor (http://mendel.imp.ac.at/pts1/PTS1predictor.jsp). Functional domains were predicted using BLASTP (https://blast.ncbi.nlm.nih.gov/Blast.cgi?PAGE=Proteins). Abb. abbreviation, PTS1 peroxisomal targeting signal 1, GFP enhanced green fluorescence protein, FL full length, 12aaC the last C-terminal 12 amino acids, Px peroxisomal, C cytosolic, nd not detected,*

* *PTS1-like signal*.

### Heterologous Localization of *G. theta* Urate Oxidase in Peroxisomes of *P. tricornutum*

*In silico* analyses predicted several proteins in *G. theta* that might be targeted to peroxisomes *via* PTS1-mediated import (refer to Table 1). For verification, heterologous *in vivo* localization studies were conducted in the diatom *P. tricornutum*, which in comparison with *G. theta* has a similar subcellular compartmentation. The diatom was used in several previous studies as a suitable model system for localization studies of cryptophyte proteins (e.g., Gould et al., 2006; Gile et al., 2015; Mix et al., 2018). However, it had to be verified whether the diatom is capable of importing cryptophyte peroxisomal matrix proteins *via* the PTS1 import pathway.

**Figure 1:**
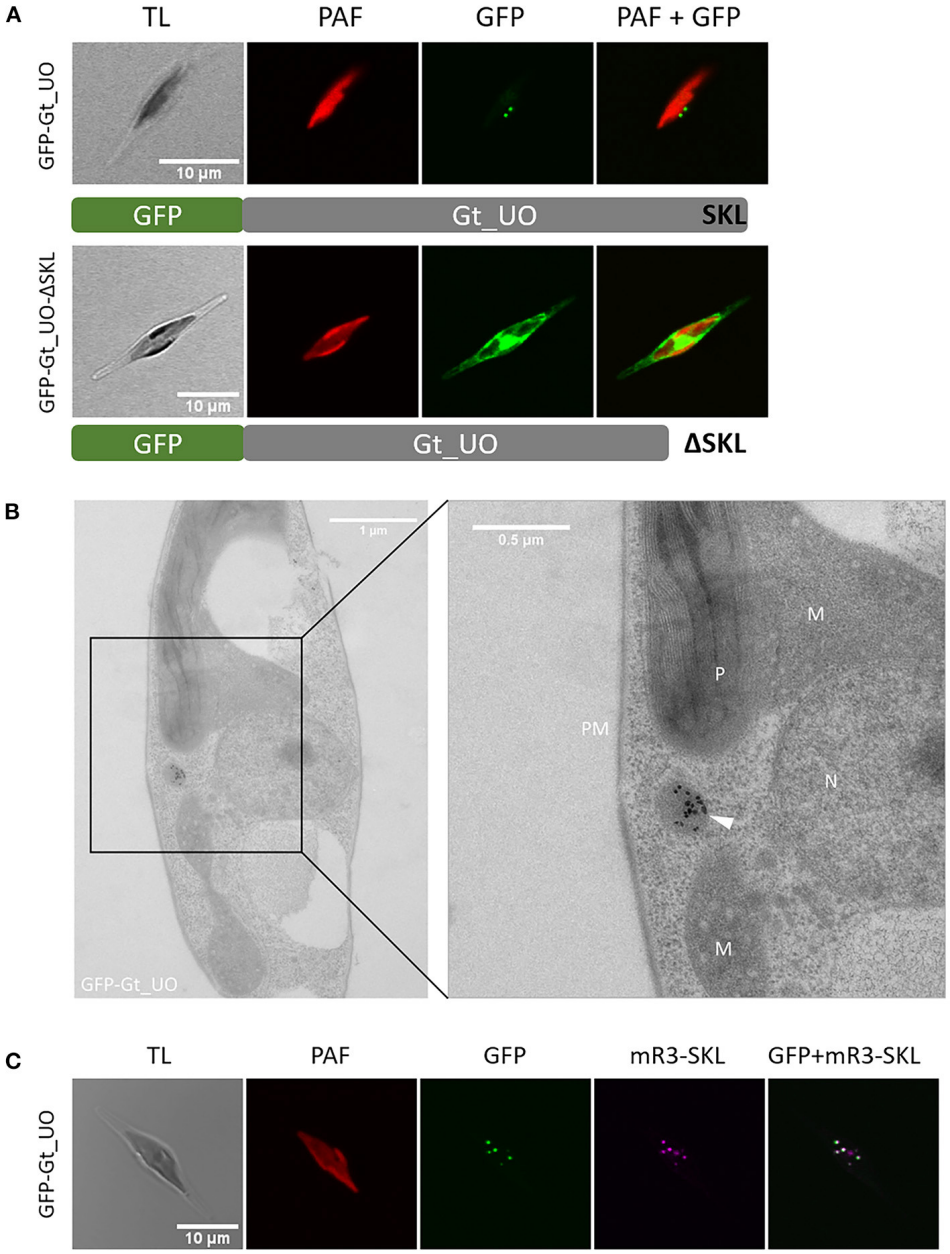
Heterologous *in vivo* localization and analysis of PTS1 function of *G. theta* urate oxidase (UO) expressed in *P. tricornutum*. **(A)** Expression of N-terminally fused GFP to the full-length Gt_UO-containing C-terminal “SKL” as PTS1 in *P. tricornutum* resulted in small dot-like structures next to the plastid. Without the C-terminal tripeptide “SKL,” GFP-Gt_UO-ΔSKL fluorescence was observed throughout the whole cell. **(B)** Immunolabeling of GFP-Gt_UO expressing *P. tricornutum* with α-GFP revealed electron-dense structures in round-shaped compartment near the plastid P, mitochondria M, and nucleus N; PM plasma membrane. **(C)** Co-expression of GFP-Gt_UO and the peroxisomal marker mRuby3-SKL (Marter et al., 2020) in *P. tricornutum* resulted in similar small dot-like structures next to the plastid. TL transmitted light, PAF plastidial autofluorescence, GFP enhanced green fluorescence protein, mR3-SKL mRuby3-SKL fluorescence protein as peroxisomal marker, scale bar in (A, C) 10 μm, scale bar in (B) overview 1 μm and 0.5 μm in section.

Gt_UO (urate oxidase) with the putative PTS1 “SKL” at its C-terminus has exemplarily been localized in full length and as a control with deleted PTS1 as a fusion protein with N-terminal GFP in *P. tricornutum*, respectively (refer to Figure 1A). Whereas expression of the GFP-Gt_UO (N-terminal GFP) resulted in a dot-like fluorescence structure typical for peroxisomes, GFP-Gt_UO-ΔSKL (N-terminal GFP, “SKL” deleted) localized to the cytoplasm of the diatom (refer to Figure 1A).

In addition to the *in vivo* localizations of Gt_UO, analyses of the GFP-Gt_UO expressing clone (Figure 1B) *via* transmission electron microscopy (TEM) using immunogold labeling on ultrathin sections were carried out. First, a *P. tricornutum* clone expressing GFP-Gt_UO was immunolabeled with α-GFP to verify the heterologous localization of GFP-Gt_UO. As shown in Figure 1B, a round-shaped electron-dense structure with 200–400 nm in diameter was marked by gold particles, indicating a compartment located next to the mitochondrion and the plastid. Similar structures were observed by McCarthy et al. (2017) and Mix et al. (2018). Further, co-localization studies with the peroxisomal marker mRuby3-SKL stably expressed by a *P. tricornutum* cell line (Marter et al., 2020) were established to fasten reliable heterologous localization. For GFP-Gt_UO, small dot-like GFP fluorescence was observed with the confocal microscopy clearly co-localizing with the mRuby3-SKL fluorescence (refer to Figure 1C).

Together with the results for the heterologously localized *G. theta* peroxins (Mix et al., 2018), these results illustrate that the peroxisomal targeting information of *G. theta* peroxins and other classical peroxisomal proteins containing a PTS1 signal is basically recognized in *P. tricornutum*, making the diatom a suitable model system for studying the localization of PTS1-containing candidates from *G. theta*.

### Specific Anti-peptide Antibody α-Gt_UO.12aaC-Labeled Peroxisomes in *G. theta* and *P. tricornutum*

**Figure 2:**
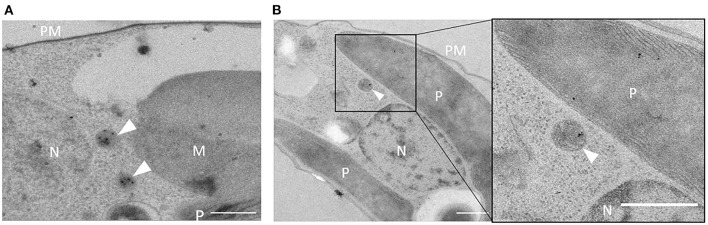
Immunolabeling with α-Gt_UO.12aaC in *G. theta* and *P. tricornutum* expressing GFP-Gt_UO. Electron-dense structures in small round-shaped compartments were immunolabeled with the specific antibody α-Gt_UO.12aaC on ultrathin sections of *G. theta* **(B)** wildtype and *P. tricornutum* **(A)** expressing GFP-Gt_UO indicating localization in peroxisomes. In *G. theta* additional labeling in the plastid could be observed. PM plasmid membrane, P plastid, N nucleus, M mitochondrion, scale bar 0.5 μm.

To confirm peroxisomal localization of Gt_UO in *G. theta*, a specific anti-peptide antibody was derived from the last 12aa of the peptide sequence, including the C-terminal PTS1 signal “SKL” (PIDGPSGYITSSASLNRSKL). In western blot analyses, specific binding of the α-Gt_UO.12aaC could be determined (refer to Supplementary Figure 1). Further, the α-Gt_UO.12aaC antibody was used for immunolabeling on ultrathin sections of *P. tricornutum* clone expressing GFP-Gt_UO and on *G. theta* wildtype sections (refer to Figure 2). Similar to the labeling with α-GFP (refer to Figure 1B), a round-shaped immunogold-labeled electron-dense structure was observed in *P. tricornutum* (refer to Figure 2A), indicating a peroxisome or a small spherical compartment and confirming the specific binding of the anti-peptide antibody. Since the specific binding of the α-Gt_UO.12aaC was confirmed on ultrathin sections in the heterologously expressing diatom and in western blot analyses with whole protein extract of *G. theta*, endogenous localization studies of Gt_UO in *G. theta* were carried out *via* TEM. Immunolabeling with α-Gt_UO.12aaC on ultrathin *G. theta* sections revealed electron-dense structures in a tiny round-shaped compartment, with some additional labeling in the plastid (refer to Figure 2B). Through bioinformatic analyses, no N-terminal signal peptide for Gt_UO was determined; therefore, it has to be analyzed further if Gt_UO indeed localizes in both peroxisome and the plastid or if the electron-dense structures in the plastid are representing an unspecific binding of the antibody.

### Co-localization Studies of *G. theta* PTS1-Containing Proteins in *P. tricornutum*

A stable transfected *P. tricornutum* cell line expressing mRuby3-SKL as a peroxisomal marker (Marter et al., 2020) was used to co-express GFP-tagged *G. theta* candidates containing putative PTS1. These include not only the peroxisome-specific UO that plays a role in purine metabolism but also APX for ROS detoxification and further enzymes participating in important metabolic pathways: vitamin K (MenB), ether phospholipid (FAR, AGPS), carbohydrate (MD, NME), amino acid (AAT), and amine metabolism (MAO). The corresponding genes were PCR amplified without introns from cDNA to ensure a correct open reading frame expression in the heterologous diatom model system. Most of the candidates were localized as full-length proteins with GFP fused N-terminally. This way, the C-terminal PTS1 signal necessary for recognition by the soluble Pex5 receptor responsible for peroxisomal import was not masked and a potential sterical hindrance was kept low. Localized proteins in different constructs are summarized in Table 1.

**Figure 3:**
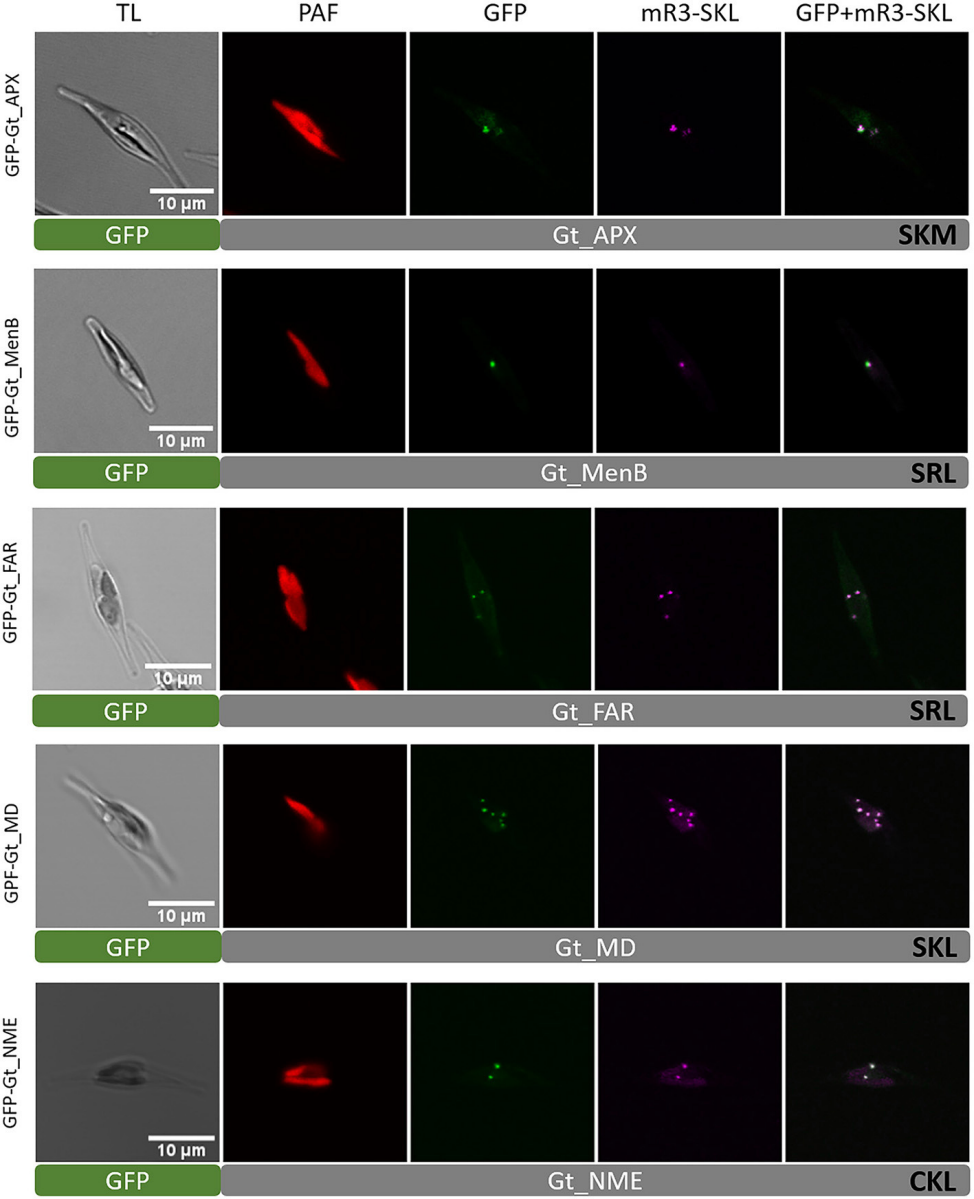
Heterologous *in vivo* co-localization of *G. theta* PTS1-containing proteins co-expressed in full length in *P. tricornutum* mR3-SKL. Heterologous localization studies of *G. theta* PTS1-containing enzymes in *P. tricornutum* expressing a peroxisomal marker (mR3-SKL). Peroxisomal localization was confirmed for APX (ascorbate peroxidase), MenB (1,4-dihydroxy-2-naphtoyl-CoA synthase), FAR (fatty acyl-CoA reductase), MD (malate dehydrogenase), and NME (NAD-dependent malic enzyme). TL transmitted light, PAF plastid autofluorescence, GFP enhanced green fluorescence protein, mR3-SKL mRuby3-SKL fluorescence protein as peroxisomal marker, scale bar 10 μm.

As mentioned above, Gt_UO was heterologously co-localized in the mRuby3-SKL expressing *P. tricornutum* strain (refer to Figure 1C). UO participates in the breakdown of purines producing H_2_O_2_ as a by-product. Although a typical peroxisomal H_2_O_2_-detoxifying catalase was not identified, bioinformatic analyses revealed putative PTS1-containing ascorbate peroxidase (APX). GFP-Gt_APX was co-localized in full-length heterologously in the *P. tricornutum* strain expressing mRuby3-SKL (refer to Figure 3) and might take over the detoxification of *G. theta* peroxisomes from ROS.

Identification of a naphthoate synthase (1,4-dihydroxy-2-naphthoyl-CoA synthase, MenB) containing a putative PTS1 in *G. theta* that is encoded by *menB* indicates the peroxisomal participation in vitamin K synthesis. GFP-Gt_MenB was co-localized with the peroxisomal marker in *P. tricornutum*, verifying peroxisomal localization of the fusion construct (refer to Figure 3). Note that GFP-Gt_MenB was also observed in bigger punctuate structures in *P. tricornutum* next to the plastid and peroxisomes not co-localizing these (refer to Supplementary Figure 6).

Peroxisomes of *G. theta* also seem to participate in ether phospholipid synthesis, as we could identify a PTS1-containing fatty-acyl-CoA reductase (FAR) and an alkyl-dihydroxyacetone-phosphate synthase (AGPS) with putative PTS1 signals. A co-localization with the peroxisomal marker was confirmed in *P. tricornutum* for GFP-Gt_FAR. The fatty alcohols produced by FAR are crucial during the synthesis of ether phospholipids since these provide the alkyl moiety for the AGPS reaction step, which generates alkyl-dihydroxyacetone phosphate (alkyl-DHAP) (Wanders and Brites, 2010; Dean and Lodhi, 2018). For the full-length Gt_AGPS protein with N-terminal GFP, a peroxisomal localization could not be confirmed. Instead, GFP-Gt_AGPS was observed throughout the cell, indicating cytosolic localization. In comparison, the GFP-AGPS.12aaC version showed a partial localization in peroxisomes and cytosol (refer to Supplementary Figure 2). Since the GFP fusion construct was highly overexpressed and due to heterologous localizations in *P. tricornutum*, these results should be interpreted with caution (refer to Discussion).

We identified two putative PTS1-containing proteins that might play a role in carbohydrate metabolism (refer to Table 1). A peroxisomal isoform of malate dehydrogenases was localized as GFP-Gt_MD in small dot-like structures, as shown by confocal microscopy. Visualized structures for GFP-Gt_MD were co-localized with the peroxisomal marker (mRuby3-SKL), indicating peroxisomal localization in *P. tricornutum*. A similar localization was observed for the *G. theta* NAD-dependent malic enzyme (NME) when localized as a GFP-Gt_NME fusion protein in *P. tricornutum* (refer to Figure 3).

An aspartate amino transferase (AAT) was detected in bioinformatic analyses containing a putative PTS1 signal. Similar to heterologous localization studies of Gt_AGPS in *P. tricornutum*, the full-length Gt_AAT with N-terminally fused GFP (GFP-Gt_AAT) was observed in the cytosol. Instead, GFP-Gt_AAT.12aaC co-localized with the peroxisomal marker expressed in *P. tricornutum* indicating that at least in the diatom, the last 12aa containing the C-terminal PTS1 tripeptide are recognized by the soluble Pex5 receptor for peroxisomal import (refer to Supplementary Figure 2).

A putative PTS1-containing monoamine oxidase (MAO) essential for amine metabolism was localized as a GFP-Gt_MAO.12aaC version in *P. tricornutum* to verify the capacity of the C-terminus for PTS1-mediated import into peroxisomes. Co-expressed with the peroxisomal marker mRuby3-SKL in *P. tricornutum*, fluorescence in small dot-like structures was observed (refer to Supplementary Figure 2). Due to overexpression in *P. tricornutum via* the nitrate reductase promoter, some GFP-labeled *G. theta* proteins showed not solely dot-like structures but seemed to localize partially in the cytosol (refer to Supplementary Figure 2).

### Factors of β-Oxidation in *G. theta* Were Localized in Mitochondria of *P. tricornutum*

It was remarkable that our bioinformatic research did not reveal any PTS1-containing candidates for peroxisomal β-oxidation. In mammals, peroxisomes and mitochondria share the degradation of fatty acids *via* β-oxidation, whereby in plants and yeast, only peroxisomal β-oxidation was reported (Poirier et al., 2006). In both organelles, acyl-CoA is produced by shortening acyl-CoA esters in similar reaction steps. We aimed to identify homologous proteins for all factors participating in β-oxidation and to analyze in which organelle or compartment β-oxidation takes place in the cryptomonad *G. theta*. However, *in silico* analyses suggest that the cryptophyte lacks peroxisome-targeted β-oxidation pathway. Heterologous *in vivo* localization studies and in-depth bioinformatic analyses of putative candidates were carried out to review these results.

In peroxisomes, the first step in β-oxidation of fatty acids is enforced by an acyl-CoA oxidase (ACOX) producing H_2_O_2_ as a by-product, whereas in mitochondria, an acyl-CoA dehydrogenase (ACAD) catalyzes the first reaction step transferring the electrons to the respiratory chain (Camões et al., 2015). Through bioinformatic analyses, we identified one candidate for ACOX (ID_147553) and five putative ACAD (ID_90066, ID_87673, ID_71272, ID_156886, and ID_113578) enzymes. All candidates were analyzed with several online prediction tools for putative targeting sequences, including mTP (mitochondrial targeting peptide) and PTS1 (refer to Materials and Methods). A mitochondrial localization was predicted for all Gt_ACOX and Gt_ACAD candidates. Interestingly, Gt_ACOX possesses “SR**S**” whereas Gt_ACAD with the protein ID_90066 contains “S**A**M” at the C-terminus. Both putative PTS1 signals distinguish in one amino acid from the known PTS1 consensus [SAC]-[KRH]-[LM]. Analyzing the last 12 C-terminal amino acids with the PTS1 prediction tool (Neuberger et al., 2003a,b), Gt_ACOX was predicted to be not targeted to peroxisomes whereby the putative PTS1 in Gt_ACAD could contribute to peroxisomal import, at least as predicted with general function (refer to Supplementary Table S3).

**Figure 4:**
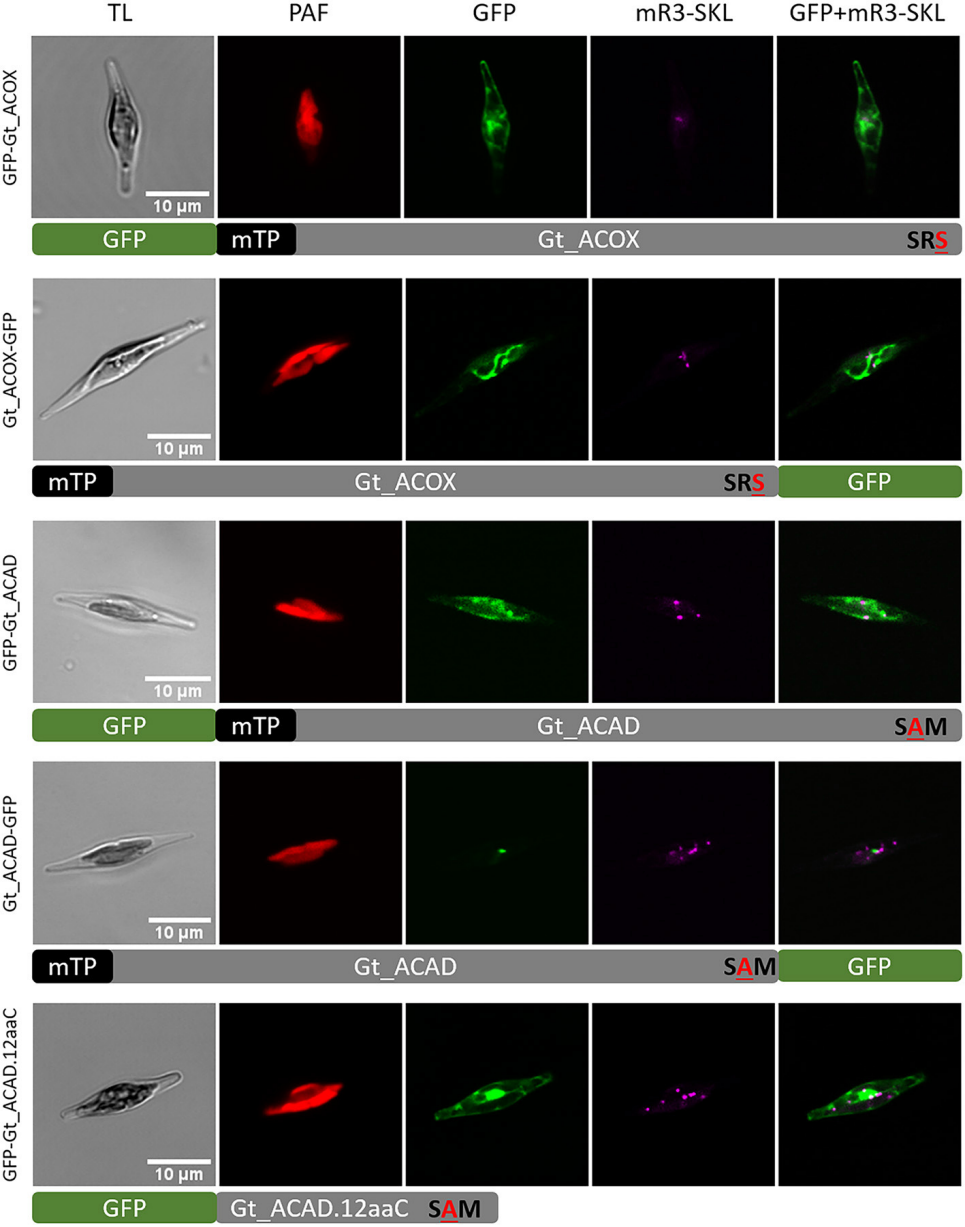
Heterologous *in vivo* co-localization studies of factors involved in the first step of fatty acid β-oxidation in *G. theta* co-expressed in *P. tricornutum* mR3-SKL. GFP-Gt_ACOX (acyl-CoA oxidase) was localized in the cytosol, whereas Gt_ACOX-GFP is targeted to the mitochondrion when localized heterologously in *P. tricornutum*. The initial mitochondrial enzyme for β-oxidation ACAD (acyl-CoA dehydrogenase) was detected in *G. theta* containing both a mTP and a non-canonical putative PTS1 signal. When expressed heterologously in *P. tricornutum*, GFP-Gt_ACAD and GFP-Gt_ACAD.12aaC were localized in the cytosol, indicating no impact of the C-terminal tripeptide on PTS1 import into peroxisomes at least in *P. tricornutum*. Expression of C-terminal GFP fused to ACAD (Gt_ACAD-GFP) in *P. tricornutum* resulted in dot-like localization not correlating the peroxisomal marker. TL transmitted light, PAF plastid autofluorescence, GFP enhanced green fluorescence protein, mR3-SKL mRuby3-SKL fluorescence protein as peroxisomal marker, scale bar 10 μm.

To determine *in vivo* location of putative peroxisomal Gt_ACOX (ID_147553) and Gt_ACAD (ID_90066), different variants of the corresponding genes were expressed as GFP fusion and localized heterologously *in vivo* in the *P. tricornutum* mRuby3-SKL cell line (Marter et al., 2020). To verify the mTP at the N-terminus, GFP was fused to the detected presequence and the full-length Gt_ACOX C-terminally. The Pre_Gt_ACOX-GFP (refer to Supplementary Figure 3) and Gt_ACOX-GFP (refer to Figure 4) were localized in elongated structures within *P. tricornutum* cells, not co-localizing with the peroxisomal marker mRuby3-SKL. Additionally, GFP was fused N-terminally to Gt_ACOX to check if the Gt_ACOX C-terminus was able to trigger peroxisomal import of the full-length Gt_ACOX *via* a potentially non-canonical PTS1-signal “SR**S**.” GFP-Gt_ACOX was localized cytosolically (refer to Figure 4 and Supplementary Figure 3), indicating that the putative PTS1 signal is not recognized *in P. tricornutum*. To verify the functionality of the C-terminal 12 amino acids (12aa), which were reported to have an impact on peroxisomal import (Neuberger et al., 2003a), GFP-Gt_ACOX.12aaC was constructed and localized in *P. tricornutum*. We observed similar localization throughout the cells compared to the GFP-Gt_ACOX version (refer to Figure 4 and Supplementary Figure 3).

Since the Gt_ACAD candidate with the protein ID_90066 contains the putative PTS1 signal “S**A**M” which was predicted in general to trigger peroxisomal import, we decided to verify the predicted localization *in vivo*. Gt_ACAD was localized in full length as GFP fusion N-/C-terminally. GFP-Gt_ACAD was localized throughout the cytosol, whereas Gt_ACAD-GFP showed in some cases dot-like structures (refer to Figure 4). However, these structures were clearly bigger than typical peroxisomes and did not co-localize with the peroxisomal marker mRuby3-SKL (refer to Figure 4).

Localized Gt_ACOX (ID_147553) and Gt_ACAD (ID_90066) candidates were analyzed bioinformatically in more detail. ACAD enzymes usually consist of an α-domain at the N-terminus, followed by a β-domain and a α-domain at the C-terminus (α-β-α), whereas ACOX has a similar structure with an additional C-terminal α-domain (α-β-α-α) (Kim and Miura, 2004). The additional α-domain results in a bigger reaction site of the protein and enables the binding of very long fatty acids, whereas ACADs usually bind long and short fatty acids. Since ACOX and ACAD enzymes are quite similar, the *in silico* differentiation can be difficult. Bioinformatic analyses of both protein sequences in PHYRE^2^ and a protein data bank (refer to Materials and methods) revealed similarities of Gt_ACOX to other known ACOX and ACAD sequences whereby for Gt_ACAD, only other ACAD sequences were identified. In any case, a doubtless, functional classification of the protein with ID 147553 is not possible at the moment.

The second and the third step in β-oxidation can either be catalyzed by bifunctional enzymes in peroxisomes or by individual enzymes in mitochondria that can be combined in a trifunctional enzyme including steps 2 to 4 (Camões et al., 2015). BLAST analyses to search for enoyl-CoA-hydratase (ECH) usually catalyzing the second step of β-oxidation resulted in the identification of three candidates in *G. theta* (ID_88345, ID_99846, ID_86481). For these candidates, no peroxisomal import was predicted; instead, each Gt_ECH candidate possesses a predicted mTP for mitochondrial targeting. Interestingly, one putative multifunctional enzyme was detected using the bifunctional enzyme from *P. tricornutum* (ID_ 55069) as query for BLAST analyses. Gt_ECH-3HCD (ID_121132) is probably catalyzing ECH and 3-hydroxyacyl-CoA dehydrogenase (3HCD) reactions, thereby combining the second and the third step of fatty acid β-oxidation. After adjusting the gene model with EST and gDNA data, no PTS but a putative mTP was predicted on gDNA, indicating solely mitochondrial targeting of Gt_ECH-3HCD. Although the mTP of Gt_ECH-3HCD is not confirmed by EST data and could not be amplified on cDNA, we decided to localize the full-length protein fused to GFP. When expressed heterologously in *P. tricornutum*, Gt_ECH-3HCD-GFP localized in the cytosol indicating the absence of a functional mTP (refer to Supplementary Figure 4).

Only one candidate, Gt_3HCD, for the individual third step of β-oxidation homologous to the bifunctional enzyme from *P. tricornutum* was identified *via* BLAST analyses with the protein ID_161810. Prediction analyses proposed mitochondrial targeting of Gt_3HCD with no putative PTS signals. Heterologous *in vivo* localization in *P. tricornutum* of Gt_3HCD-GFP resulted in elongated structures within *P. tricornutum* typical for mitochondria localized proteins (refer to Supplementary Figure 4).

For the fourth step in fatty acid β-oxidation, a 3-ketoacyl-CoA-thiolase (KCT) is required. Peroxisomal isoforms contain either a PTS1 or a PTS2 (Carrie et al., 2007; Lee et al., 2009; Gonzalez et al., 2011), and mitochondrial isoforms can also be integrated in the trifunctional enzyme combining steps 2 to 4 (Camões et al., 2015). Our bioinformatic analyses revealed a putative mitochondrial Gt_KCT (ID_159886) with no PTS that is homologous to a subunit of a trifunctional enzyme. Due to a transcript, encoding an open reading frame not supported by ESTs or transcript data, we decided to use the presequence of Gt_KCT including the predicted mTP for analyzing the targeting prediction of the N-terminus *via* GFP localization studies. In *P. tricornutum*, Pre-Gt_KCT-GFP localized in elongated structures around the plastid indicating a mitochondrial localization (refer to Supplementary Figure 4).

## Discussion

### How Reliable Are Heterologous Localization Studies?

This study is based on the heterologous localization studies of *G. theta* proteins co-expressed with a peroxisomal marker in *P. tricornutum*. Since the diatom *P. tricornutum* is capable of PTS1 but not PTS2 import (Gonzalez et al., 2011), localization studies of peroxisomal matrix proteins are limited to putative PTS1-containing proteins. Mix et al. (2018) showed that the diatom *P. tricornutum* is capable of sorting the peroxisome-specific Gt_Pexs with putative transmembrane domains (TMDs) to the peroxisomal membrane (Gt_Pex2, Gt_Pex10, Gt_Pex11-1, Gt_Pex11-2, Gt_Pex14, Gt_Pex16, Gt_Pex19-1, and Gt_Pex22), proposing *P. tricornutum* as a suitable model organism for heterologous localization studies. Here, we aimed to give new insights into metabolic capacity of *G. theta* peroxisomes focusing on heterologous co-localization studies of PTS1-containing proteins.

PTS1-containig Gt_UO was exemplarily used to prove the PTS1-functionality as a targeting signal of heterologously expressed proteins in *P. tricornutum*. Heterologous expression of the full-length Gt_UO with N-terminal GFP (GFP-Gt_UO) was co-localizing with the peroxisomal marker in small-dot like structures in *P. tricornutum* (refer to Figure 1A). Additionally, electron-dense structures were observed in small round-shaped organelles with 200–400 nm in diameter indicating peroxisomes (refer to Figures1B, 2) and the deletion of the C-terminal PTS1 “SKL” relocated the Gt_UO-ΔSKL to the cytosol (refer to Figure 1C). These results demonstrated that a least the most abundant PTS1 signal “SKL” triggers the PTS1-mediated import of heterologously expressed proteins in the diatom.

However, some PTS1-containing proteins of *G. theta* did not co-localize with the peroxisomal marker when expressed heterologously in *P. tricornutum*. Gt_AGPS (“SHL”) and Gt_AAT (“SKL”) were located dominantly in the cytosol when expressed as a full-length protein, even though the last C-terminal 12aa were imported into peroxisomes in *P. tricornutum* when fused to the C-terminus of GFP (refer to Supplementary Figure 2). These results indicate that the last 12aa at the C-terminus of heterologously expressed fusion constructs were recognized by the endogenous Pt_Pex5 receptor responsible for PTS1 import. Probably, the PTS1 signal of some FL proteins is not available for the PTS1 receptor Pt_Pex5 due to steric arrangement of the tertiary structure of the eGFP fusion protein. If so, localizations should be interpreted carefully, since the intracellular protein location in *G. theta* could differ from the findings of the heterologous localization studies in *P. tricornutum*. Nevertheless, as the predicted localization of most *G. theta* proteins was proven to be correct in co-localization studies and was widely supported by detailed *in silico* analyses of the overall protein sequences, we strongly propose *P. tricornutum* as a suitable model organism for heterologous *in vivo* localization studies of *G. theta* proteins.

### Peroxisomal Metabolism in *G. theta*

**Figure 5:**
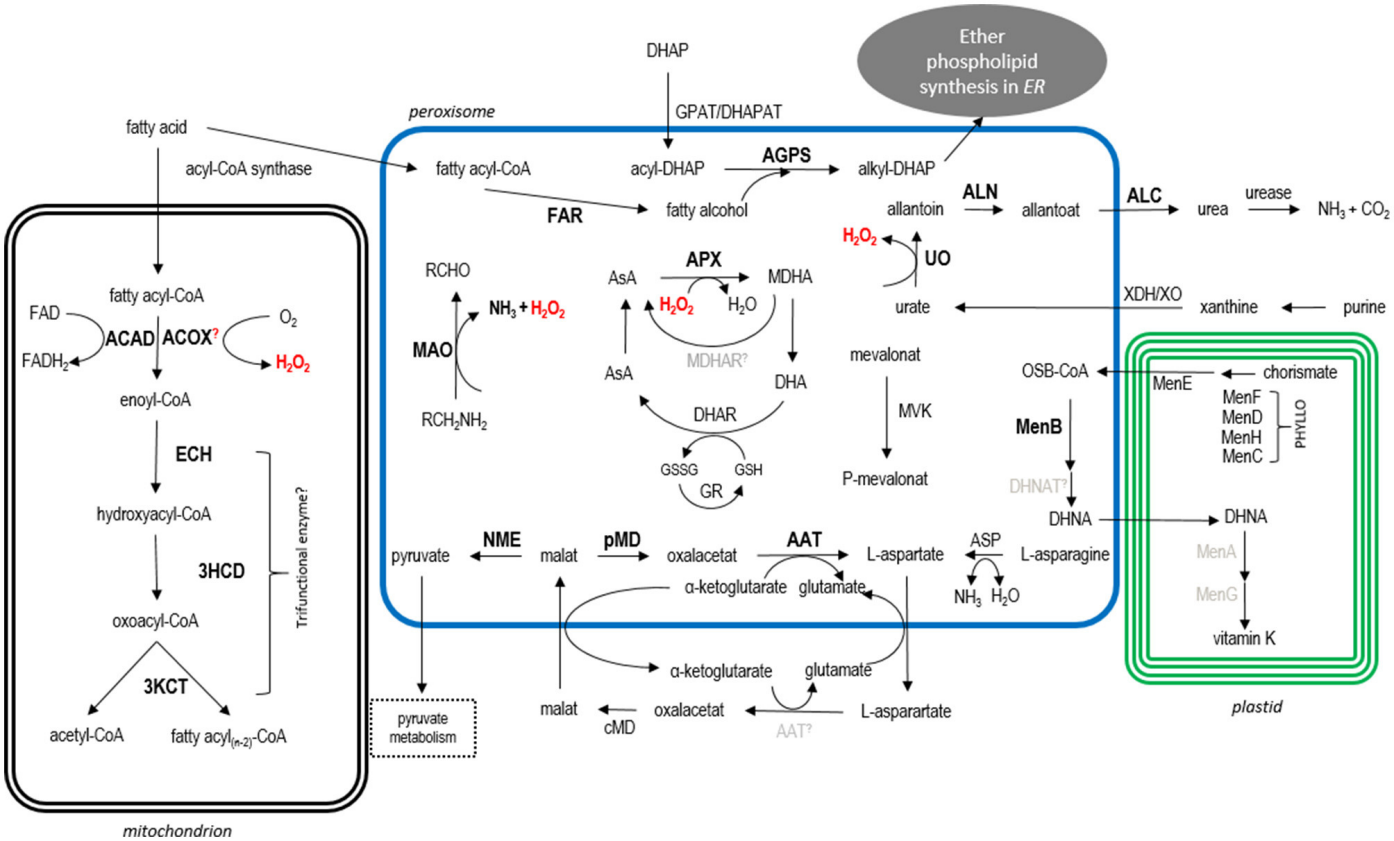
Model of the predicted metabolic pathways in the cryptomonad *G. theta*. For details see main text. Abbreviations: ACAD acyl-CoA dehydrogenase, ACOX acyl-CoA oxidase, ECH enoyl-CoA hydratase, 3HCD 3-hydroxyacyl-CoA dehydrogenase, 3KCT 3-Ketoacyl-CoA thiolase, FAR fatty acid reductase, DHAP dihydroxyacetone phosphate, GPAT/DHAPAT glycerolphosphate acyltransferase/dihydroxyacetone phosphate acyltransferase, AGPS alkyl-dihydroxyacetone phosphate synthase, MAO monoamine oxidase, AsA ascorbate, APX ascorbate peroxidase, MDHA monodehydroascorbate, MDHAR MDHA reductase, DHA dehydroascorbate, DHAR DHA reductase, GR glutathione reductase, XDH/XO xanthine dehydrogenase/oxidase, UO urate oxidase, ALN allantoinase, ALC allantoicase, ASP asparaginase, AAT amino aspartate transferase, c/pMD cytosolic/peroxisomal malate dehydrogenase, NME NAD-dependent malic enzyme, PHYLLO consisting of MenF, MenD, MenH, MenC; MenB DNC synthetase, MenA phytyltransferase/polyprenyltransferase, MenG methyltransferase, MVK mevalonate kinase. Bold localized proteins, black bioinformatically predicted location (manually detected PTS1/PTS2-like containing proteins), gray not identified in *G. theta*; further, not localized PTS1-candidates are not included in this model.

Bioinformatic analyses and *in vivo* localization studies provided a broad overview of the predicted metabolism of peroxisomes in the cryptomonad *G. theta*. Although some of the heterologous localization studies initially gave inconclusive results and thus have to be interpreted with caution (see above), the majority of our *in silico* targeting predictions were shown to be correct upon expression of *G. theta* candidate proteins as GFP fusions in *P. tricornutum*. Together with our previous study on the localization of Pex factors in *G. theta* (Mix et al., 2018), our presented work strongly suggests the presence of metabolically active peroxisomes in the cryptophyte. This conclusion is also supported by electron microscopic evidence marking the peroxisomal protein Gt_UO in a small spherical electron-dense structure most likely resembling a peroxisome in the cytoplasm of *G. theta* (refer to Figure 2B). In the following, we will discuss the possible metabolic processes that probably take place in the *G. theta* peroxisomes and how these might be connected to the metabolic network in the entire cell (refer to Figure 5).

### Gt_APX Might Be Involved in Peroxisomal H_2_O_2_ Detoxification in *G. theta*

In catabolic processes such as peroxisomal β-oxidation, purine degradation, and monoamine desamination, reactive H_2_O_2_ is produced as a by-product and has to be detoxified quickly before the cell is harmed. A typical peroxisomal enzyme for ROS detoxification is catalase which usually serves as a peroxisomal marker (Duve and Baudhuin, 1966; Ghosh and Hajra, 1986). In *P. tricornutum* and *C. reinhardtii*, a catalase for H_2_O_2_ detoxification was localized *in vivo* to peroxisomes whereby CAT1 of *C. reinhardtii* contains a non-canonical PTS1 “GCL” (Gonzalez et al., 2011; Kato et al., 2021). In *G. theta*, bioinformatic analyses did not reveal a homolog gene encoding a catalase. However, beside two isoforms predicted to be acting in the plastid, a further isoform of L-ascorbate peroxidase (Gt_APX) with the C-terminal tripeptide “SRM” was co-localized in peroxisomes of the diatom *P. tricornutum* (refer to Figure 3).

Ascorbate peroxidase catalyzes the H_2_O_2_-dependent oxidation of L-ascorbate to monodehydroascorbate (MDHA) releasing H_2_O and is an important key player in the ascorbate (AsA) – glutathione (GSH) cycle. MDHA is further reduced to AsA by a MDHA reductase (MDHAR). Alternately, MDHA can spontaneously disproportionate to AsA and dehydroascorbate (DHA). Using GSH as a cofactor, DHA is recycled to AsA by DHA reductase producing the oxidized glutathione-disulfide (GSSG). For regeneration of the oxidized GSH, GSSG is reduced by GSH-reductase (GR) (Shigeoka et al., 2002; Ishikawa and Shigeoka, 2008; Tamaki et al., 2021).

Enzymes participating in AsA-GSH cycle were identified by screening the genome database of *G. theta*. A scheme of the putative metabolic peroxisomal processes in *G. theta* including the AsA-GSH cycle is presented in Figure 5. Peroxisomal localization was confirmed in heterologous localization studies for Gt_APX (refer to Figure 3). A homolog encoding a MDHAR was not detected *via* bioinformatic analyses in *G. theta*. Most likely, MDHA disproportionates spontaneously to DHA which might be further reduced by Gt_DHAR (ID_153349) back to AsA. Interestingly, the predicted Gt_DHAR contains not only a nonapeptide “RLLLVLPLF” at the N-terminus, which has similarities to the PTS2, but also a signal peptide, proposing dual targeting to peroxisomes and the plastid. BLAST and KEGG analyses revealed a homolog to GR in *G. theta* with the protein ID_83774. This predicted protein is encoded with the C-terminal tripeptide “SRV” that could trigger peroxisomal import according to the PTS1 predictor (Neuberger et al., 2003a,b). However, “SRV” is not a classical PTS1 signal, and thus, its targeting function in the cryptophyte remains elusive. Nevertheless, “SRV” function as a PTS1 signal was validated in *Arabidopsis thaliana* (Reumann et al., 2012).

### Gt_UO and Gt_MAO Produce H_2_O_2_ as By-Product

The typical peroxisomal enzyme urate oxidase (UO) produces H_2_O_2_ as a by-product by oxidizing urate to 5-hydroxyisorate that is further converted to allantoate. UO is an important enzyme in purine degradation and recycling of nitrogen as well as glyoxylate in higher plants (*A. thaliana* and legumes) and in some Apicomplexa (*Nephromyces* and *Cardiosporidium*) (Todd et al., 2006; Hauck et al., 2014; Paight et al., 2019).

Purine degradation starts with purine desamination to xanthine, which is further converted to urate by xanthine dehydrogenases/oxidases (XDH/XO). XDH/XO activities can be combined in xanthine oxidoreductase (XOR) (Nishino et al., 2008). UO oxides urate to 5-hydroxyisorate, which is further converted to (S)-allantoin (Kahn and Tipton, 1998; Lamberto et al., 2010). (S)-Allantoin is converted by an allantoinase (ALN) to allantoate which is then processed by allantoicase to urea and ureidoglycolate. In the following step, urea can be recycled to CO_2_ and ammonia by urease. Alternatively, either ureidoglycolate is processed to glyoxylate or urea excreted as waste.

Here, we report a partial peroxisomal purine degradation pathway in *G. theta* resulting most likely in recycling of ammonia. Bioinformatic analyses of homologs involved in purine degradation revealed cytosol-located purine desaminases (not included in Figure 5) producing xanthine that is further converted to urate by Gt_XDH/XO with the protein ID_164776 lacking both a predictable PTS1 and PTS2 (refer to Figure 5). In *G. theta*, peroxisomal localization was confirmed for Gt_UO (refer to Figures 1, 2) producing H_2_O_2_ as by-product. A putative Gt_ALN with the protein ID_67814 contains the classical PTS1 “AKL” as C-terminal tripeptide. Heterologous *in vivo* localization studies in *P. tricornutum* revealed cytosolic localization for GFP-Gt_ALN (full length), whereas the last 12aa at the C-terminus fused to GFP (GFP-Gt_ALN.12aaC) localized in small dot-like structures resembling peroxisomes (refer to Supplementary Figure 5). No peroxisomal localization could be confirmed for the putative peroxisomal isoform Gt_ALC (ID_71482) expressed as full length or 12aa.C version with N-terminal GFP fusion with the putative PTS1 “SK**V**” in *P. tricornutum*. The PTS1-like signal “SK**V**” is deviating from the typical PTS1 pattern in the last amino acid which might affect the PTS1 recognition in *P. tricornutum* by the endogenous PTS1 receptor Pex5. A second putative candidate for Gt_ALC (ID_71482) is annotated in KEGG and might be located in the cytosol of *G. theta* (refer to Supplementary Figure 5). The homolog of urease with the protein ID_116849 in *G. theta* contains no predicted PTS1 but “KLKRLARGL” as N-terminal nonapeptide reminding of a PTS2 signal. However, *G. theta* PTS2-containing proteins cannot be localized heterologously in *P. tricornutum* because the PTS2 import pathway is missing in the diatom (Gonzalez et al., 2011; Mix et al., 2018).

It has to be noted that also, non-canonical PTS1 and PTS2 could contribute to peroxisomal import in *G. theta* peroxisomes which have not been identified *via in silico* predictions and might not be recognized in heterologous localization studies in *P. tricornutum*. For example, localization studies of non-canonical PTS in *C. reinhardtii* imply that PTS signals in certain microalgae might have a wider variation in amino acid composition than reported in higher plants (Lauersen et al., 2016; Kato et al., 2021).

Nevertheless, according to the heterologous localization studies, the purine degradation in *G. theta* seems to be partially located in peroxisomes. Moreover, bioinformatic analyses propose the degradation of urea resulting in reutilization of CO_2_ and ammonia. Green freshwater algae were shown to utilize allantoin as nitrogen source, supporting our results (Prasad, 1983). Upregulation of enzymes involved in purine degradation in *Chlamydomonas reinhardtii* proposed that the pathway is similar to higher plants with ammonia as end-product rather than urea (Park et al., 2015).

Another enzyme participating in H_2_O_2_ formation in *G. theta* peroxisomes was classified as monoamine oxidase (MAO) by signal search containing the C-terminal tripeptide “SRM.” Heterologous co-localization studies of the last 12aa of Gt_MAO in *P. tricornutum* confirmed peroxisomal localization (refer to Supplementary Figure 2). MAO is a flavoprotein with the cofactor FAD and uses oxygen for deamination of amines resulting in the corresponding ketone or aldehyde, H_2_O_2_, and ammonia (Gaweska and Fitzpatrick, 2011; Abad et al., 2013).

Taken together, *in vivo* localization studies and bioinformatic analyses propose at least partial compartmentalization of H_2_O_2_ formation during purine degradation or amine deamination and its rapid detoxification by AsA-GSH cycle in the peroxisomes of the cryptomonad *G. theta* (refer to Figure 5).

### Carbohydrate and Amino Acid Metabolism in *G. theta* Peroxisomes

Besides the H_2_O_2_ formation and detoxification, several additional *G. theta* PTS1-containing proteins were identified and localized heterologously in peroxisomes of *P. tricornutum*, co-expressed with mRuby3-SKL as peroxisomal marker. The summarized putative metabolic map in *G. theta* is presented in Figure 5. *G. theta* peroxisomes seem to participate in carbohydrate metabolism since a PTS1-containing malate dehydrogenase and a NAD-dependent malic enzyme, which are involved in malate metabolism, co-localized with mRuby3-SKL in *P. tricornutum* peroxisomes.

Malate dehydrogenases (MD) convert malate to oxaloacetate and are therefore important key players in several metabolic processes (Kroth et al., 2008; Davis et al., 2017). In *G. theta*, three MD isoforms are annotated in KEGG and were analyzed *via* bioinformatics. We identified a cytosolic isoform (Gt_cMD) with the ID_163842, a predicted mitochondrial isoform (Gt_mMD) with the ID_154188, and a peroxisomal isoform (Gt_pMD) with the protein ID_64327 containing the typical “SKL” tripeptide at the C-terminus. Peroxisomal localization was confirmed for Gt_pMD in heterologous localizations studies in *P. tricornutum* (refer to Figure 3). Furthermore, Gt_pMD and Gt_cMD might participate in the glyoxylate cycle or the malate-aspartate shuttle that requires future in-depth analyses. Most likely, malate is synthetized in the cytosol or mitochondria and is transported into *G. theta* peroxisomes *via* the malate-aspartate shuttle (mitochondrial malate-aspartate shuttle is not included in Figure 5). One bioinformatically identified malate synthase (MS) with the protein ID_97605 was localized as GFP-Gt_MS.12aaC in the cytosol (data not shown), despite containing a non-canonical PTS1-like signal “SRK” which did not trigger PTS1-mediated import in *P. tricornutum*. Additionally, Gt_MS could be imported *via* a predicted N-terminal mTP into mitochondria. It has been proposed that N-terminal targeting signals might be more competitive in comparison with C-terminal PTS1 (Kunze and Berger, 2015; Stehlik et al., 2020). Nevertheless, dual targeting of Gt_MS cannot be excluded and should be analyzed in the future studies (not shown in Figure 5). Mitochondrial isoform Gt_mMD could participate in the TCA cycle (Sweetlove et al., 2010) (not shown in Figure 5) which has to be analyzed in further studies. Malate can be additionally converted by NAD(P)-dependent malic enzyme (ME) to pyruvate (Tronconi et al., 2008), contributing to the pyruvate metabolism. In *G. theta*, a NAD-dependent ME (Gt_NME) containing “CKL” as PTS1 was detected and peroxisomal localization was confirmed *via* heterologous localization studies in *P. tricornutum* (refer to Figure 3). The carbohydrate metabolism in the diatoms *P. tricornutum* and *T. pseudonana* was elucidated in detail (Kroth et al., 2008; Davis et al., 2017). Compared to the cryptomonad *G. theta*, the diatoms possess peroxisomal and mitochondrial isoforms for MD but only a mitochondrial NME (Kroth et al., 2008; Davis et al., 2017). In *C. reinhardtii*, two MD isoforms containing a PTS2 (MDH1 and MDH2) were *in vivo* localized to peroxisomes (Hayashi and Shinozaki, 2012; Hayashi et al., 2015; Lauersen et al., 2016). A knockout of *MDH2* in *C. reinhardtii* affected peroxisomal β-oxidation of fatty acids, indicating that lipid metabolism is connected to a functional MDH2 in peroxisomes (Kong et al., 2018).

Previously known as glutamate oxaloacetate transaminase (GOT), aspartate amino transferase (AAT) converses aspartate with ketoglutarate to glutamate and oxaloacetate (Kirsch et al., 1984). For full functionality of AAT, pyridoxal 5'-phosphate is important, which is bound to AAT (Kirsch et al., 1984; Toney, 2014). AATs play usually an important role in the mitochondrial malate-aspartate shuttle and are also involved in breakdown of amino acids. For the identified Gt_AAT (ID_158574) with “SKL” as PTS1, a heterologous localization in *P. tricornutum* peroxisomes was confirmed (refer to Supplementary Figure 2). Further bioinformatic analyses of aspartate metabolism in *G. theta* revealed a putative asparaginase (Gt_ASP, ID_121809) with non-canonical “PRL” at the C-terminus indicating a putative PTS1 signal. In general, ASP hydrolyzes L-asparagine to L-aspartate releasing ammonia. Peroxisomal localization was confirmed for several proteins containing a “PRL” as the PTS1 signal in higher plants (*A. thaliana*), yeast (*S. cerevisiae*), and also an ascomycete pathogen (*Colletotrichum higginsianum*) (Reumann, 2004; Reumann et al., 2012; Nötzel et al., 2016; Robin et al., 2018). Whether the C-terminal tripeptide “PRL” also triggers peroxisomal PTS1-dependent import in *P. tricornutum* has to be validated in the future studies.

### Vitamin K Synthesis in the Cryptomonad *G. theta*

Heterologous *in vivo* localization studies of PTS1-containing proteins of *G. theta* in *P. tricornutum* partially confirmed peroxisomal localization of the *in silico* identified 1,4-dihydroxy-2-naphtoyl-CoA (DHNA-CoA) synthase (MenB) encoded by *guithdraft_85732* in *G. theta* (refer to Figure 3). Next to peroxisomal GFP-Gt_MenB, deviating localization in bigger dot-structures was observed in *P. tricornutum* not co-localizing the peroxisomal marker which should be addressed in the future studies (refer to Supplementary Figure 6). MenB is one of the central enzymes involved in vitamin K synthesis. In photosynthetic organisms, vitamin K1 (phylloquinone) is an important electron carrier in photosystem I (Del Río, 2013; Cenci et al., 2018). Hence, the *de novo* biosynthesis of phylloquinone is an essential pathway in higher plants and photosynthetic organisms. Here, we investigated bioinformatically the enzymes involved in vitamin K synthesis and summarized the results in Figure 5.

In nine consecutive reaction steps, phylloquinone is synthesized from chorismate which is the product from shikimate pathway. In the first four steps, chorismate is converted to *o*-succinyl benzoate by plastid-targeted enzymes. The proteins involved in phylloquinone biosynthesis are functional similar to the enzymes involved in menaquinone (vitamin K2) biosynthesis and are therefore called Men enzymes. The first four steps are combined in a multifunctional protein referred as PHYLLO combining MenF, MenD, MenH, and MenC (Gross et al., 2006, 2008; Cenci et al., 2018). In higher plants, the isochorismate synthase (MenF) catalyzing the first reaction can also be encoded by individual genes enabling the independent regulation of other metabolic branches such as the synthesis of salicylic acids required for plant defense (Gross et al., 2006, 2008). Through bioinformatic analyses, we identified a putative Gt_PHYLLO protein with the protein ID_72208 in *G. theta* combining MenD, MenH, and MenC, which was predicted to localize to the plastid. Gt_MenF (ID_72109) is annotated in KEGG as menaquinone-specific isochorismate synthase. Additionally, a putative Gt_MenH (ID_72176) encoding gene was identified in the same gene cluster together with ID_72208 and ID_72109. If the identified Gt_MenF and Gt_MenH encoding individual genes or part of the PHYLLO cluster has to be addressed in future studies, the activation of *o*-succinyl-benzoate by an *o*-succinyl-benzoyl-CoA ligase (MenE) was determined in the peroxisomes in *A. thaliana* by peroxisomal acyl-CoA activating enzyme (AAE14, At1g30520) with non-canonical PTS1 “S**S**L” which might be also dually targeted to plastids (Kim et al., 2008; Babujee et al., 2010; Del Río, 2013 chapter 12 by S. Reumann). According to the localization prediction tools, the homologous Gt_MenE (ID_139711) contains no PTS-like signals and localizes to the plastid. It should be noted that only the very last C-terminal part of the putative Gt_MenE is predicted to function as *o*-succinyl benzoate-CoA ligase. After the activation of *o*-succinyl-benzoate, MenB catalyzes the naphtoate ring formation. MenB is encoded by a single gene and contains a conserved PTS2 in all putative orthologs in higher plants analyzed so far (Reumann et al., 2007; Babujee et al., 2010). Contrary to higher plants, Gt_MenB contains a more abundant C-terminal “SRL” and is imported into peroxisomes in a PTS1-dependent manner (refer to Supplementary Figure 6). Interestingly, in *P. tricornutum*, a “SKL”-containing homologous to MenB encoding gene (PHATRDRAFT_bd371) was identified with KEGG annotations proposing peroxisomal involvement in vitamin K biosynthesis in the diatom. DHNA-CoA thioesterase (DHNAT) produces DHNA by releasing CoA. In *Arabidopsis thaliana*, two genes encoding DHNAT were identified, both containing PTS1-signals, and were demonstrated to localize in plant peroxisomes (Reumann et al., 2009; Widhalm et al., 2012). No homologous gene encoding the specific DHNAT was identified in *G. theta* in our bioinformatic approach. Instead, we could identify several acyl-CoA thioesterases with putative hotdog-containing domains (refer to Supplementary Table S5), which were reported to be involved in the synthesis of phylloquinones (Dillon and Bateman, 2004; Widhalm et al., 2009). If one of these candidates is indeed a DHNAT participating in phylloquinone synthesis in *G. theta*, which has to be analyzed in future studies, the last two steps of vitamin K1 biosynthesis take place in the plastids of higher plants. MenA (DHNA phytyltransferase) attaches the phytyl chain to the naphthoate ring and MenG methylates the precursor to phylloquinone. No homologs encoding Gt_MenA or Gt_MenG were identified in *G. theta*. Usually, Men enzymes are located in one cluster on genomic level (Gross et al., 2008). The identified putative PHYLLO cluster (ID_72208 and ID_72109) should be examined in the future studies and investigated in respect to its catalyzed reactions. Finally, a vitamin-K-epoxide reductase (VKOR) was identified in *G. theta* (ID_106868, not included in Figure 5) and is predicted to localize in the plastidial membrane comparable with the thylakoid located VKOR in *A. thaliana* (Tie and Stafford, 2008; Feng et al., 2011).

### Ether Phospholipid Synthesis

Bioinformatic analyses identified a putative peroxisomal Gt_AGPS, which catalyzes one of the peroxisomal reactions during ether phospholipid synthesis. Heterologous *in vivo* co-localization studies in *P. tricornutum* are inconclusive, since a cytosolic localization was observed for the full-length protein (GFP-Gt_AGPS) whereas GFP-AGPS.12aaC co-localized with the peroxisomal marker (refer to Supplementary Figure 2). Therefore, a peroxisomal localization of Gt_AGPS in *G. theta* can be assumed (refer to Figure 5). AGPS exchanges the acyl group with an alkyl group of acyl-dihydroxyacetone phosphate (acyl-DHAP), which is acetylated in advance by glycerolphosphate acyltransferase (GPAT) or dihydroxyacetone phosphate acyltransferase (DHAPAT) (Hajra and Bishop, 1982; Hajra and Das, 1996; Dean and Lodhi, 2018). Inspections with KEGG in *G. theta* propose two Gt_GPAT/DHAPAT isoforms (ID_139343 and ID_163230) with 5-6 transmembrane domains without a predicted PTS or mTP. Additionally, the second putative Gt_GPAT/DHAPAT (ID_163230) is predicted to be secreted (N-terminal signal peptide) and is most likely involved in triacylglycerol (TAG) synthesis. Therefore, Gt_GPAT/DHAPAT (ID_139343) is probably activating DHAP and is attached to peroxisomal membrane which requires more analyses in the future (refer to Figure 5). Besides DHAP as an intermediate, AGPS also requires fatty alcohols that provide the alkyl residue. Fatty alcohols are catabolized by FARs. Gt_FAR homolog was identified as PTS1-containing enzyme and co-localized heterologously in peroxisomes of *P. tricornutum* (refer to Figure 3). Together with Gt_AGPS, Gt_FAR is involved in first steps in ether phospholipids synthesis which is completed in the endoplasmic reticulum (ER) (refer to Figure 5).

### β-Oxidation of Fatty Acids Takes Place in Mitochondria of *G. theta*

In eukaryotic cells, β-oxidation of fatty acids can take place either in mitochondria or in peroxisomes. The enzymatic reactions are similar varying in single enzymes and/or complexes. Peroxisomal β-oxidation starts with ACOX, whereas ACAD is most likely to be present in mitochondrial pathway. Both enzymes, ACOX and ACAD, form a trans-double bond in the acyl-CoA thioester. Steps 2–3 are catalyzed by bifunctional enzymes in peroxisomes; in mitochondria, individual enzymes but also trifunctional enzymes were observed combining steps 2–4 (Camões et al., 2015). These reactions include the addition of a water molecule by ECH (step 2), dehydrogenation by 3HCD (step 3), and the final acylation by 3KCT (step 4) resulting in the formation of an acetyl-CoA and acyl-CoA shortened by two carbon atoms.

In plants and fungi, β-oxidation is exclusively peroxisomal, whereas in mammals, both enzymatic pathways (mitochondria and peroxisomes) are presented. In examined algae so far, several dispositions according to the location of enzymes participating in β-oxidation were observed. In the charophyte *Mougeotia*, an exclusively peroxisomal β-oxidation was reported (Stabenau et al., 1984). In the diatom *P. tricornutum*, PTS1-containing isoforms for every step in peroxisomal β-oxidation of fatty acids were determined (Gonzalez et al., 2011). Additionally, a mitochondrial isoform of KCT (ID_28068) is annotated in *P. tricornutum*, and a mitochondrial PtMACAD1 encoded by *Phatrdraft3_J11014* was identified (Jallet et al., 2020) proposing that the degradation of fatty acids *via* β-oxidation is taking place in mitochondria and peroxisomes in the diatom. Dual localization of β-oxidation enzymes was observed in the chlorophyte *Eremosphaera* and in several Prasinophytes (Winkler et al., 1988; Stabenau et al., 1989), similar to the situation in mammals. Additionally, solely mitochondrial β-oxidation was observed in the xanthophyte *Bumilleriopsis* and in *Cyanidium* (Gross et al., 1985; Gross, 1989). In *C. reinhardtii*, an ACOX encoded by *CrACX2* was co-localized with peroxisomal CrMDH2 in peroxisomes, indicating at least partial peroxisomal β-oxidation of fatty acids (Kong et al., 2017).

Remarkably, *in silico* analyses did not reveal any homologs to enzymes that participate in β-oxidation containing the typical PTS1 signal consisting of [SAC]-[KRH]-[LM] in *G. theta*. Candidates for the first step of β-oxidation (ACOX/ACAD) were identified by bioinformatics with non-canonical C-terminal tripeptide reminding of a PTS1 signal (“SR**S**”/“S**A**M”) and additionally containing a mTP at the N-terminus, proposing dual targeting to peroxisomes or mitochondria of *G. theta*, respectively. However, heterologous co-localization studies in *P. tricornutum* expressing the peroxisomal marker mRuby3-SKL proposed mitochondrial localization for both *G. theta* enzymes (refer to Figure 4 and Supplementary Figure 3). For further differentiation, phylogenetic analyses and/or *in vitro* assays of the *G. theta* ACOX and ACAD should be conducted.

The following steps in β-oxidation are either catalyzed by individual or multifunctional enzymes, depending on the organism and the intracellular location of this pathway. In *G. theta*, several individual homologs for ECH, the second step in β-oxidation, were identified with predicted mTPs. Gt_ECH (ID_161810) was localized to the mitochondria of *P. tricornutum* (refer to Supplementary Figure 4). One putative multifunctional Gt_ECH-3HCD (ID_121132) probably combining steps 2–3 was predicted with a mTP after gene model correction with gDNA sequence. However, the putative mTP of ID_121132 was not confirmed by EST data and could not be amplified on cDNA. Nevertheless, we localized Gt_ECH-3HCD without the predicted mTP to the cytosol of *P. tricornutum* (refer to Supplementary Figure 4). It has to be still analyzed in more detail, if Gt_ECH-3CH is indeed a cytosol located protein and if it is able to catalyze both reaction steps mentioned above. Additionally, the presequence of Gt*_*KCT (ID_159886) catalyzing the fourth and last step in β-oxidation was observed in mitochondria when expressed heterologously as a GFP fusion in *P. tricornutum* underpinning the solely mitochondrial localization of β-oxidation in *G. theta* (refer to Supplementary Figure 4). If Gt_KCT in *G. theta* is a part of the multifunctional enzyme or if it is catalyzing, an individual reaction should be examined in the future studies.

Based on the *in silico* and heterologous *in vivo* localization studies of *G. theta* isoforms, β-oxidation takes place in the mitochondria (refer to Figure 5). It cannot totally be excluded, that some reaction steps are peroxisomal or dually localized to peroxisomes and mitochondria, because Gt_ACOX/Gt_ACAD contain non-canonical PTS1-like sequences (“SR**S**”/”S**A**M”), which might trigger peroxisomal import in *G. theta* and are possibly not recognized in *P. tricornutum*. Interestingly, in the ACOX homolog of *C. reinhardtii*, no PTS signal was detected but mCherry-CrACX2 was co-localized with a peroxisomal marker indicating that CrACX2 is imported *via* a non-PTS1 pathway (Kong et al., 2017). Besides, both Gt_ACOX and Gt_ACAD contain a N-terminal mTP which could compete on *in vivo* targeting in *G. theta* (Kunze and Berger, 2015; Stehlik et al., 2020). Additionally, all *G. theta* homologs that might participate in β-oxidation were analyzed for PTS2 signals. None of the identified *G. theta* homologs to β-oxidation participating enzymes contain the PTS2 signal [RK]-[LVIQ]-X_5_-[HQ]-[LAF] (Petriv et al., 2004). Moreover, non-canonical PTS2 sequences might exist in those *G. theta* sequences that might not be able to achieve their potential signaling effect in the diatom system because the PTS2 import pathway is absent in *P. tricornutum* (Gonzalez et al., 2011). However, taken into consideration the confirmed peroxisomal localization of PTS1-containing *G. theta* proteins and studies by Mix et al., 2018 in the heterologous system, the localizations in *P. tricornutum* are in most cases reliable. For further analyses, Gt_ACOX and Gt_ACAD should be characterized in more detail directly in *G. theta*, e.g., by immunolabeling with specific antibodies on *G. theta via* TEM.

## Conclusions

In this study, we present *in silico* and experimental proof for the presence of metabolically active peroxisomes in *G. theta*. Interestingly, in the cryptophyte, these organelles most likely lack enzymes for fatty acid β-oxidation, which is the classical metabolic pathway of peroxisomes. Our results suggest that β-oxidation in *G. theta* seems to be exclusively mitochondrial, which is in contrast to the most other organisms studied so far, although it cannot be excluded that at least some β-oxidation enzymes are targeted to peroxisomes by non-canonical signals or are dually localized. Moreover, *G. theta* peroxisomes are devoid of catalase, but have kept their ROS detoxification capability to shield the cell from harmful byproducts of metabolic reactions taking place in *G. theta* peroxisomes: e.g., purine degradation and deamination. Additionally, *G. theta* peroxisomes play an important role in amino acid metabolism, the ascorbate-glutathione cycle, vitamin K, and ether phospholipid synthesis, being connected to a multitude of other biochemical pathways in the cryptophyte cell. Thus, peroxisomes are essential compartments in the complex metabolic network of *G. theta*. In future studies, the PTS1 and PTS2 sequence motifs in *G. theta* should be analyzed in more detail since at least some algal PTSs seem to be more diverse compared to the known motifs of higher plants.

## Data Availability Statement

The datasets presented in this study can be found in online repositories. The names of the repository/repositories and accession number(s) can be found in the article/Supplementary Material.

## Author Contributions

DM conceived this study together with JV and UM. JV, A-KM, TH, and DM did the experimental work and *in vivo* localization studies in *P. tricornutum*. DM conducted the *in silico* analyses. TH performed the immunolabelling and electron microscopy analysis. The manuscript was written by JV, DM, and UM. All authors read and approved the manuscript.

## Funding

This work was funded by the Deutsche Forschungsgemeinschaft (DFG, German Research Foundation) — 409750538 (MO 3129/3-1).

## Conflict of Interest

The authors declare that the research was conducted in the absence of any commercial or financial relationships that could be construed as a potential conflict of interest.

## Publisher's Note

All claims expressed in this article are solely those of the authors and do not necessarily represent those of their affiliated organizations, or those of the publisher, the editors and the reviewers. Any product that may be evaluated in this article, or claim that may be made by its manufacturer, is not guaranteed or endorsed by the publisher.
