# Supplementary figures and images for: Inferred Subcellular Localization of Peroxisomal Matrix Proteins of Guillardia theta Suggests an Important Role of Peroxisomes in Cryptophytes
Source: Front Plant Sci. 2022 Jun 16;13:889662. doi: 10.3389/fpls.2022.889662 (PMC9244630; doi:10.3389/fpls.2022.889662)

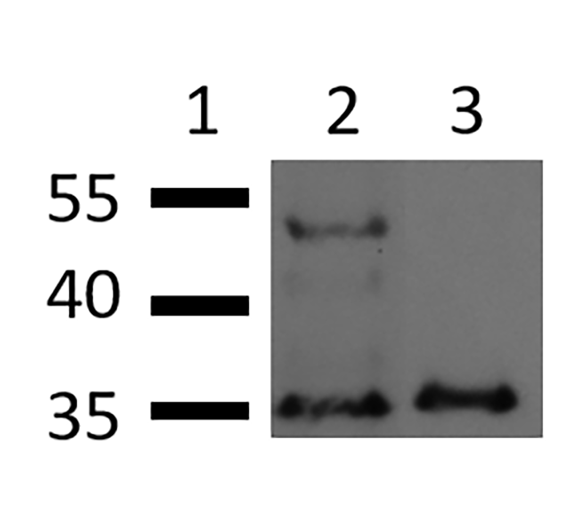

Supplement: Supplementary file 6 [file Image_1.TIF]

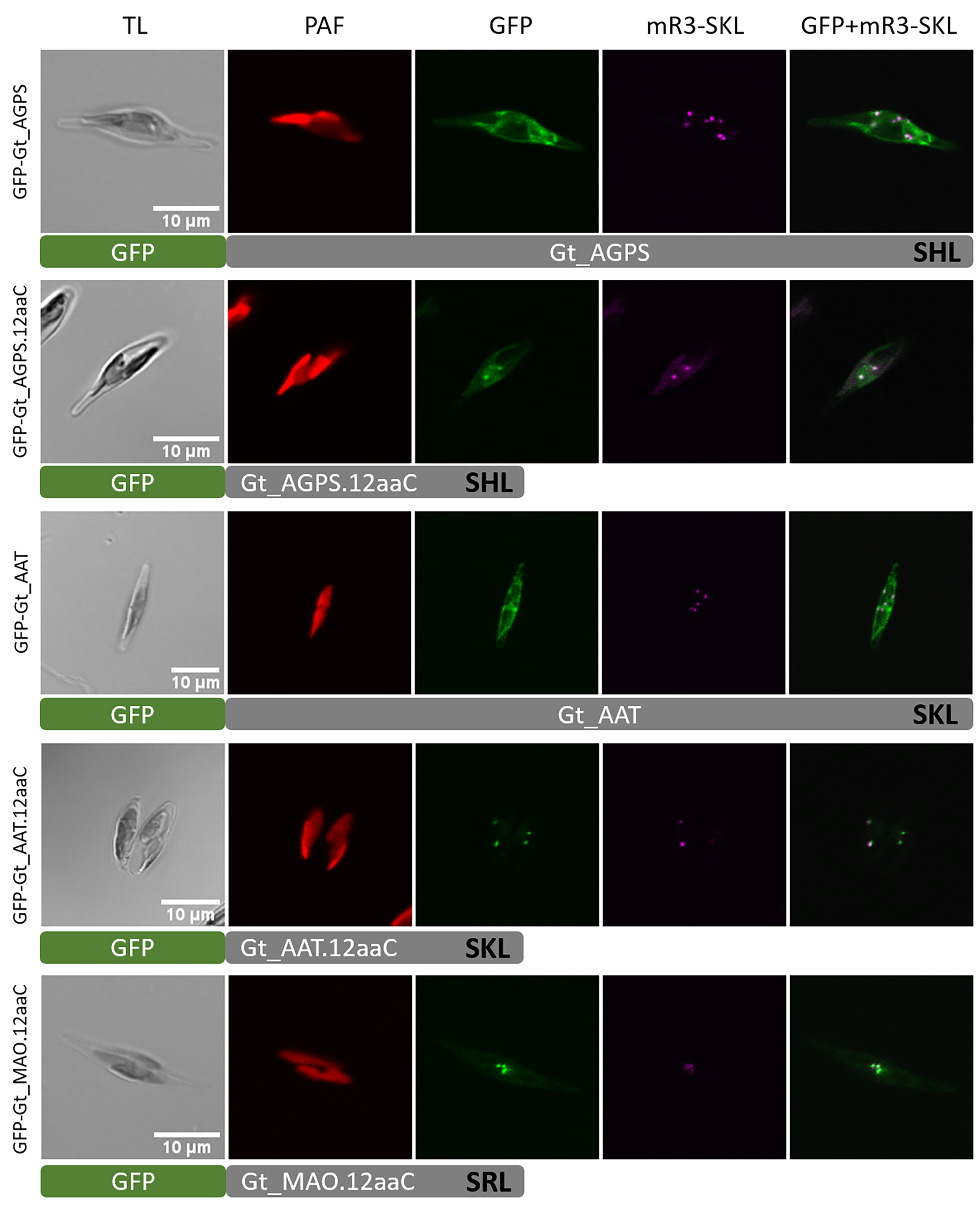

Supplement: Supplementary file 7 [file Image_2.TIF]

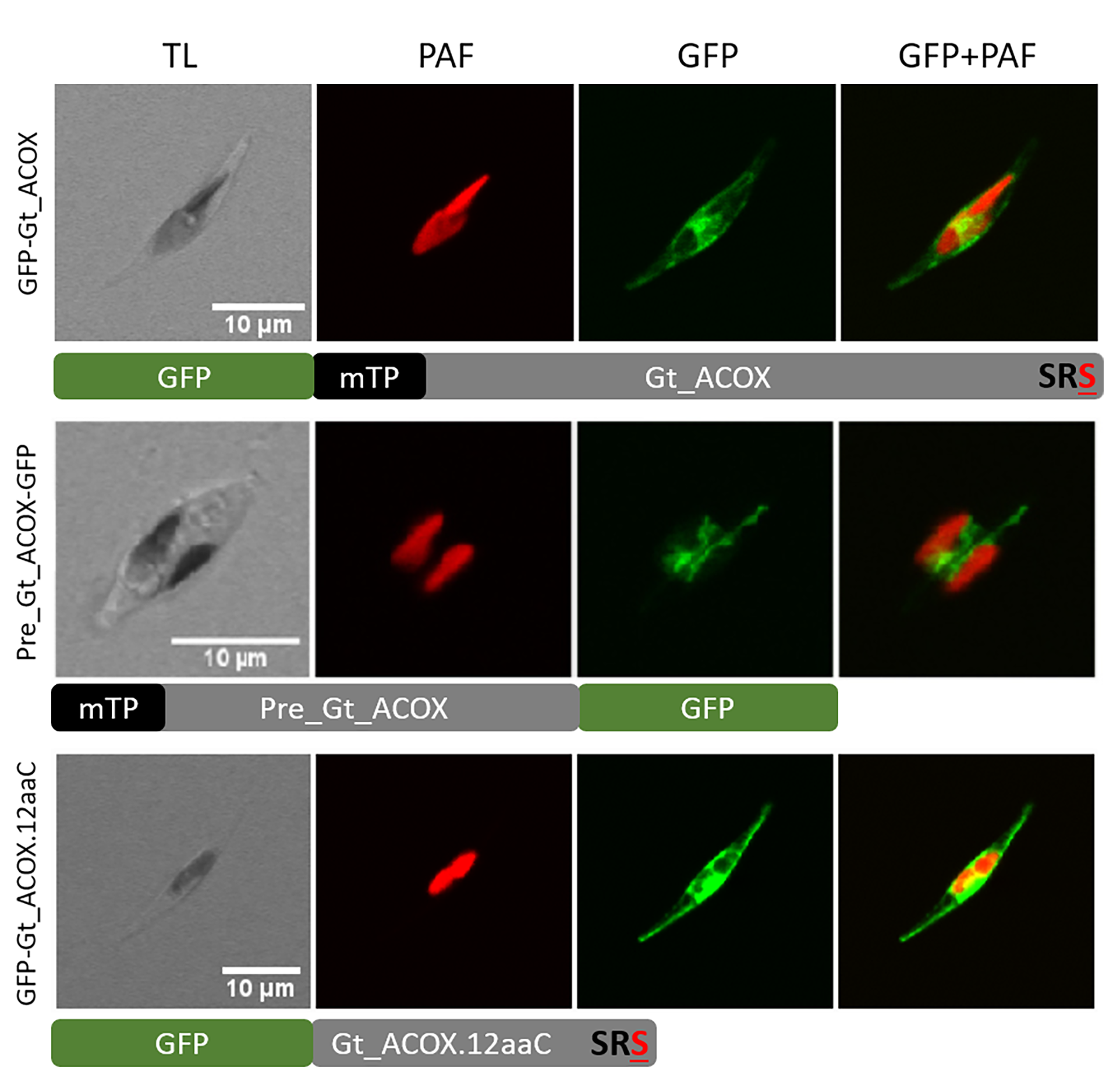

Supplement: Supplementary file 8 [file Image_3.TIF]

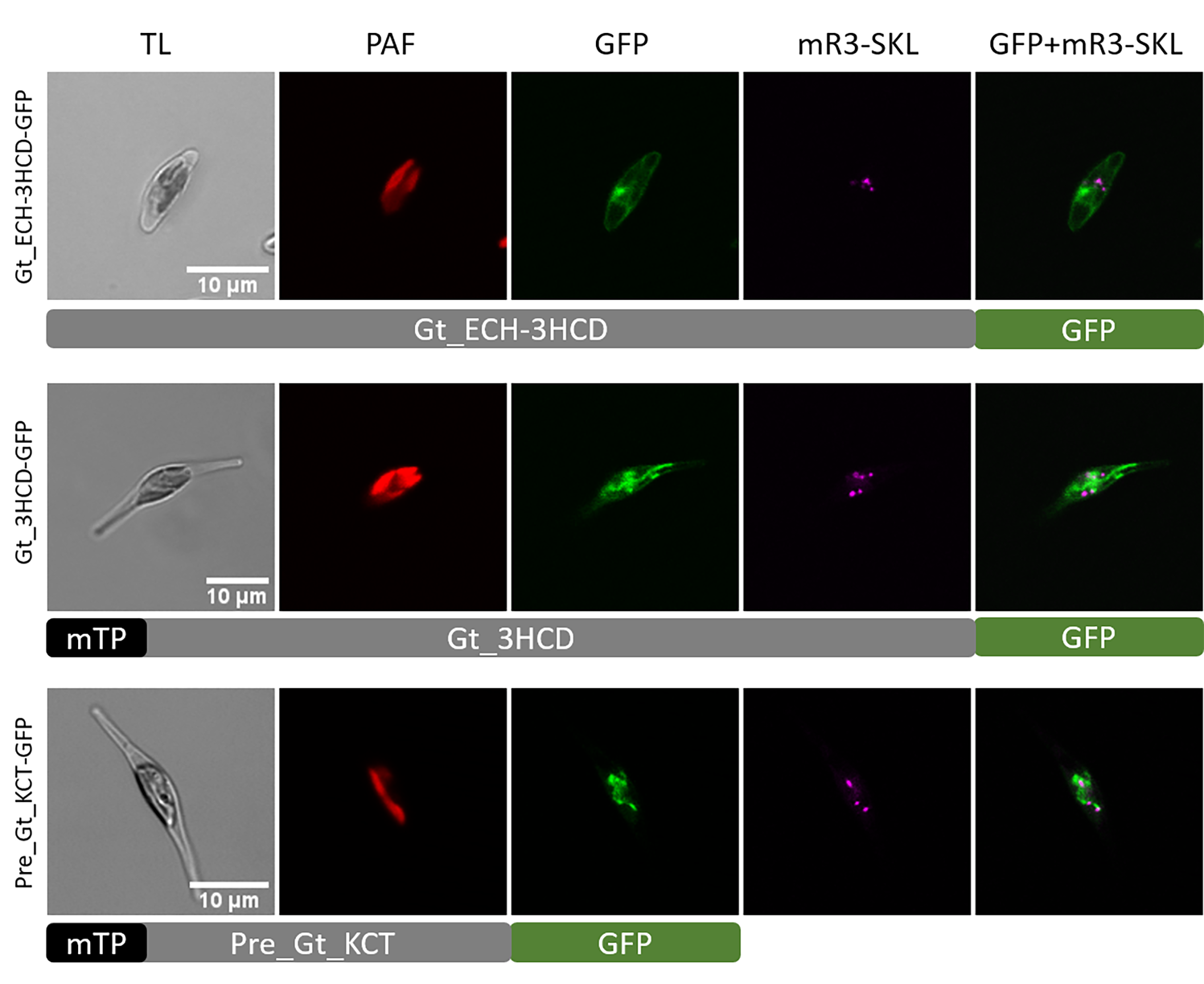

Supplement: Supplementary file 9 [file Image_4.TIF]

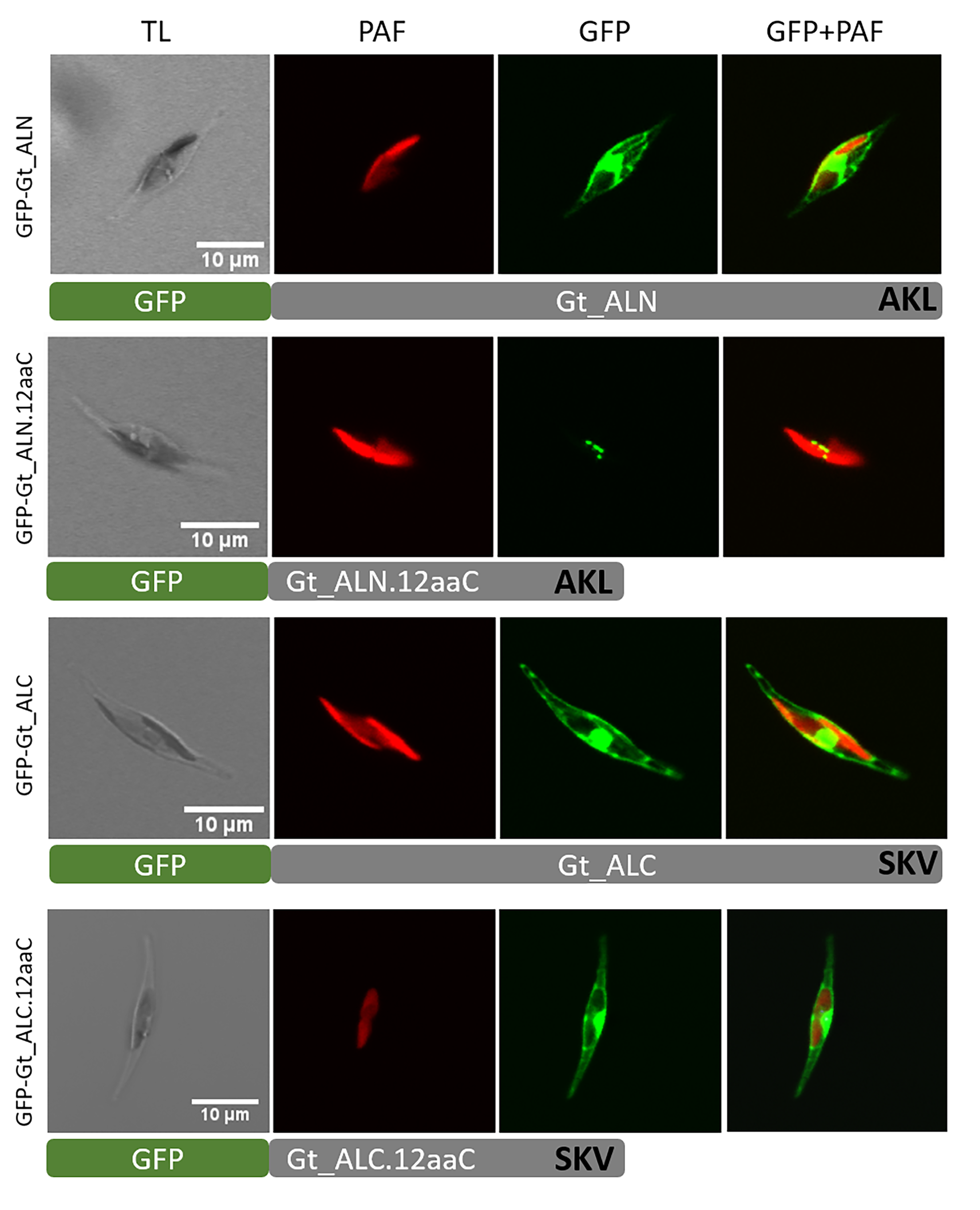

Supplement: Supplementary file 10 [file Image_5.TIF]

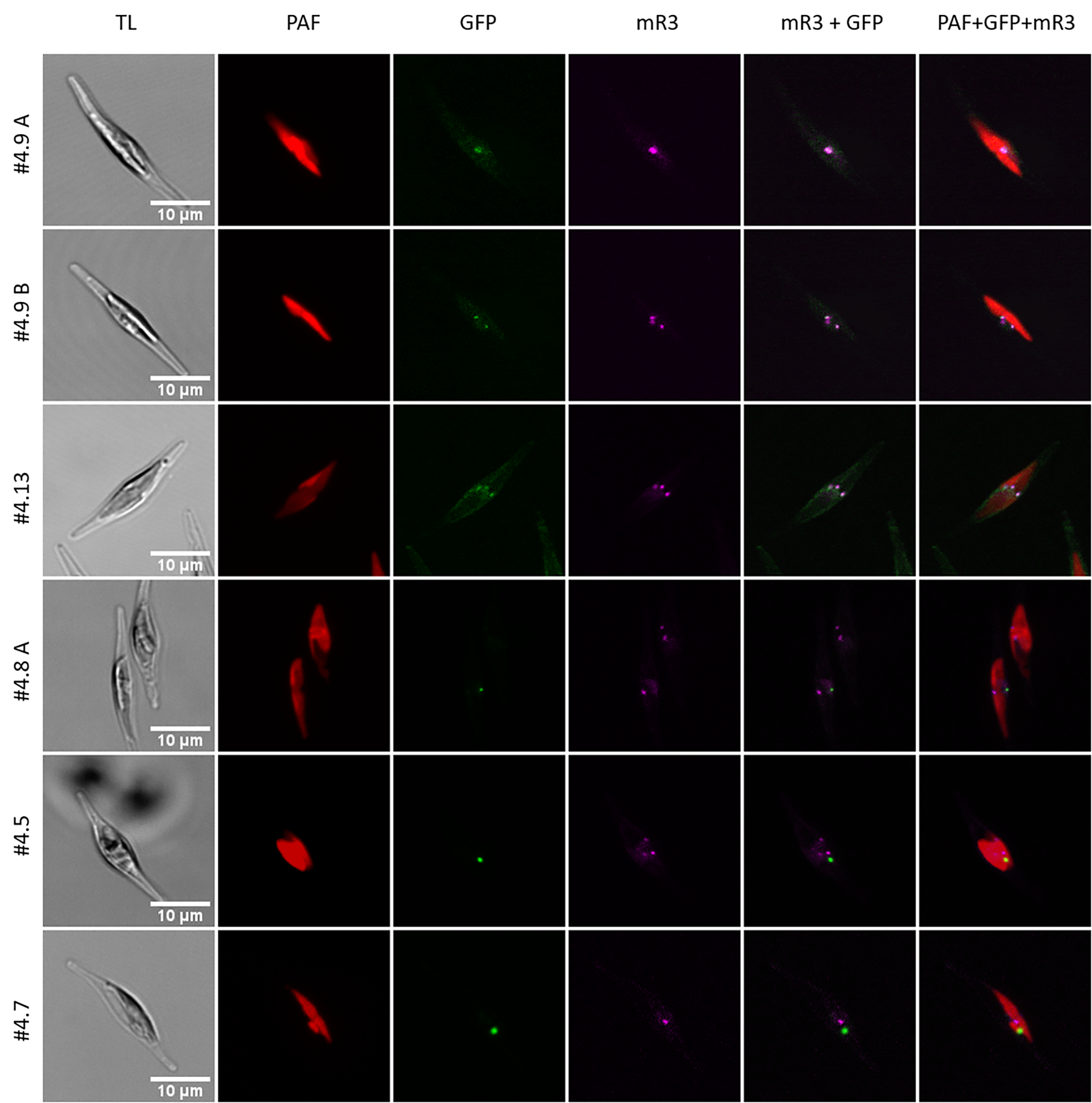

Supplement: Supplementary file 11 [file Image_6.TIF]
